# Resident bacteria contribute to opportunistic infections of the respiratory tract

**DOI:** 10.1371/journal.ppat.1009436

**Published:** 2021-03-19

**Authors:** Yifan Wu, Yongqiang Wang, Huiming Yang, Qian Li, Xiaoxia Gong, Guozhong Zhang, Kui Zhu

**Affiliations:** 1 National Center for Veterinary Drug Safety Evaluation, College of Veterinary Medicine, China Agricultural University, Beijing, China; 2 Key Laboratory of Animal Epidemiology of the Ministry of Agriculture, College of Veterinary Medicine, China Agricultural University, Beijing, China; 3 Beijing Key Laboratory of Detection Technology for Animal-Derived Food Safety and Beijing Laboratory for Food Quality and Safety, China Agricultural University, Beijing, China; University of Tubingen, GERMANY

## Abstract

Opportunistic pathogens frequently cause volatile infections in hosts with compromised immune systems or a disrupted normal microbiota. The commensalism of diverse microorganisms contributes to colonization resistance, which prevents the expansion of opportunistic pathogens. Following microbiota disruption, pathogens promptly adapt to altered niches and obtain growth advantages. Nevertheless, whether and how resident bacteria modulate the growth dynamics of invasive pathogens and the eventual outcome of such infections are still unclear. Here, we utilized birds as a model animal and observed a resident bacterium exacerbating the invasion of *Avibacterium paragallinarum* (previously *Haemophilus paragallinarum*) in the respiratory tract. We first found that negligibly abundant *Staphylococcus chromogenes*, rather than *Staphylococcus aureus*, played a dominant role in *Av*. *paragallinarum*-associated infectious coryza in poultry based on epidemic investigations and *in vitro* analyses. Furthermore, we determined that *S*. *chromogenes* not only directly provides the necessary nutrition factor nicotinamide adenine dinucleotide (NAD^+^) but also accelerates its biosynthesis and release from host cells to promote the survival and growth of *Av*. *paragallinarum*. Last, we successfully intervened in *Av*. *paragallinarum*-associated infections in animal models using antibiotics that specifically target *S*. *chromogenes*. Our findings show that opportunistic pathogens can hijack commensal bacteria to initiate infection and expansion and suggest a new paradigm to ameliorate opportunistic infections by modulating the dynamics of resident bacteria.

## Author summary

There is an urgent need for novel intervention strategies and techniques to address the increasing dissemination of multidrug-resistant Gram-negative bacterial pathogens. More importantly, secondary bacterial infections are common in clinical practice, whereas the growth dynamics of each individual in such coinfections are still complicated and elusive. In the current study, we first identified *Staphylococcus* spp., especially negligibly abundant *S*. *chromogenes*, facilitating the pathogenesis of *Av*. *paragallinarum*, a Gram-negative bacterium responsible for severe and acute avian respiratory disease worldwide. Furthermore, we developed therapeutic strategies using specific antibiotics against *Staphylococcus* spp. to relieve clinical symptoms and reduce *Av*. *paragallinarum*-associated infections in chickens. These results show that implementation of a proper intervention strategy can prevent opportunistic infections by regulating the microbiota and elucidate the development of alternative approaches for treating Gram-negative pathogenic bacterial infections.

## Introduction

Microbial communities coevolved with hosts are abundant on the surface of their bodies and the respiratory and gastrointestinal tracts. The communities consist of trillions of commensal microorganisms, also termed the microbiota, periodically interacting with various pathogenic invaders. Although resident bacteria in the mucosa are necessary to hosts to exclude exogenous pathogens [1, 2], they may be exploited by pathogens to impair hosts under certain circumstances. For instance, *Bacteroides* species, comprising a predominant genus in the intestinal tract, promote the replication and expression of virulence genes of *Escherichia coli* [3, 4]. Additionally, *Staphylococcus aureus* can prevent clearance by macrophages through the secretion of α-toxin, resulting in the expansion of opportunistic pathogens [5]. Similarly, collateral damage to the intestinal microbiota by oral antibiotics and invasive viruses renders hosts susceptible to *Clostridium difficile* [6] and *Haemophilus* spp. [7], respectively. Similar to the well-characterized modulation of bacterial dynamics in the gut, we suspected that opportunistic bacteria might also utilize resident bacteria to initiate colonization and invasion in the respiratory tract.

In contrast to the presence of abundant nutrients in the intestinal tract, resident bacteria in the respiratory tract commonly suffer from a shortage of energy sources in healthy individuals [8], especially microorganisms such as *Haemophilus* spp. that require rich nutrition. *Haemophilus* spp. are commensal Gram-negative bacteria in both humans and animals, serving as opportunistic pathogens for volatile diseases [9, 10]. Normally, the abundance of NAD^+^ in the respiratory tract of healthy individuals is insufficient for their survival and growth [11–13] because the limited resource of NAD^+^ is subtly modulated by hosts to maintain homeostasis [13, 14]. Hence, we hypothesized that the increased level of NAD^+^ is a prerequisite for the subsequent infections associated with *Haemophilus* spp. Despite the structural anatomical differences between mammals and birds, similar syndromes and potential bird-borne zoonoses have been described [15–20]. Therefore, it is convenient to employ birds to explore the contribution of resident bacteria to such infections. Infectious coryza (IC) is an acute respiratory infection of poultry worldwide caused by *Avibacterium paragallinarum*, previously known as *Haemophilus paragallinarum*, with subsequent consequences, including poor growth performance of growing chickens and a remarkable decrease in egg production (reduced by 10%-40%) [21, 22]. Interestingly, the principle of the satellitism test has been utilized for decades to isolate and identify infections associated with *Haemophilus* spp. and *Av*. *paragallinarum* [23]. Nevertheless, it is still unknown whether and how *Staphylococcus* spp. contribute to the pathogenesis of such infections *in vivo*.

In the present work, we observed negligibly abundant *Staphylococcus chromogenes* directly facilitating the survival of *Av*. *paragallinarum* in the upper respiratory tract (URT) of chickens. In addition, host cells in the presence of *S*. *chromogenes* and *Av*. *paragallinarum* were forced to increase the synthesis and release of NAD^+^, which in turn promoted the replication of *Av*. *paragallinarum*. In addition, antimicrobial agents targeting *S*. *chromogenes* significantly relieved clinical symptoms and reduced the burden of *Av*. *paragallinarum* in chickens. Our results indicate that resident bacteria in the respiratory tract can promote the invasion and replication of opportunistic pathogens, and modulation of commensal bacterial dynamics may be an alternative strategy to prevent and control such infections.

## Results

### Extensive spread of multi-drug resistant *Av*. *paragallinarum* in China

**Fig 1:**
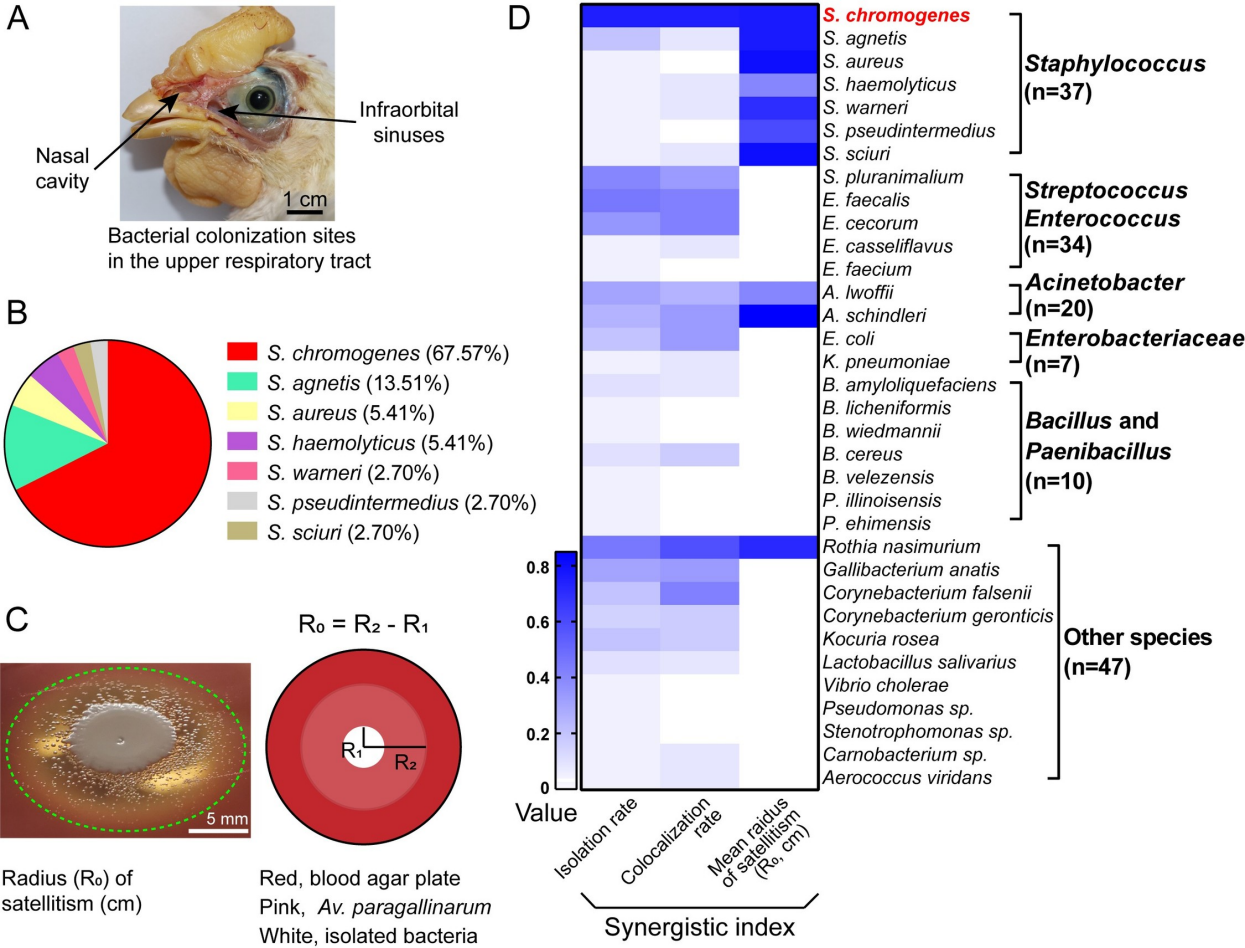
*S*. *chromogenes* is closely related to *Av*. *paragallinarum* infection. (A) Bacterial samples were collected from the nasal cavity and infraorbital sinuses of 20 birds with clinical symptoms. (B) Distribution of *Staphylococcus* spp. (C) Representative image of the satellitism of *Av*. *paragallinarum* on blood agar plates after cocultivation for 48 h. (D) Custom synergistic indexes, including the isolation rates (0–1), colocalization rates (0–1), and the mean radii of satellitism (cm).

To investigate persistent *Haemophilus* infection in the respiratory tract of birds, we first launched an epidemiological investigation by online questionnaires to reveal the prevalence of IC. We found that the rate of IC outbreaks was 59.2% (61/103) in poultry farms from 11 provinces in China (S1 Fig), which was highly associated with the breeds and origins of growing birds (S1 and S2 Tables). Additionally, we observed that the implementation of vaccinations was not able to provide enough protection against *Av*. *paragallinarum* infection. Therefore, we collected samples of the nasal cavity and infraorbital sinuses from 20 birds with clinical signs of IC (Fig 1A) because *Av*. *paragallinarum* commonly colonize such anatomic interconnected sites [21, 24]. In total, we obtained 173 isolates from 20 birds, of which 10.4% (18) were *Av*. *paragallinarum*. One isolate belonged to the type serovar B, two isolates were not typeable, and the other 15 isolates were serovar C based on the DNA sequence homology of the outer membrane protein HMTp210 (S2A Fig) [25]. However, previous reports show that *Av*. *paragallinarum* serovars A and B are dominant in China [26, 27]. Subsequently, these 18 *Av*. *paragallinarum* isolates were divided approximately into two clusters according to core-genome SNP-based phylogenetic trees (S2A Fig). Notably, *Staphylococcus* was the most prevalent genus in the other isolates, accounting for 23.87% (37/155), among which *S*. *chromogenes* displayed the highest proportion (67.57%, 25/37) (Fig 1B). Furthermore, we found that 94.6% (35/37) of *Staphylococcus* isolates were coagulase-negative *Staphylococcus* (CoNS) (Fig 1B), consistent with the phenomenon that many CoNS are associated with chronic rhinosinusitis [28]. Last, most *Av*. *paragallinarum* and *S*. *chromogenes* phenotypically showed multidrug resistance through whole-genome sequencing and antimicrobial susceptibility tests (S4 and S5 Tables and S4A and S4B Fig).

To better understand the contribution of resident bacteria to *Haemophilus*-associated infections, we performed further analyses based on custom synergistic indexes, including the rates of isolation and colocalization and the radii of satellitism on blood agar plates (Fig 1C and 1D). Two *Av*. *paragallinarum* isolates were randomly selected for satellitism tests, and the radii were measured after 48 h (Figs 1C, 1D and S3A and S3A and S3B). We observed that most bacteria in the *Staphylococcus* genus, particularly *S*. *chromogenes*, promoted the growth of *Av*. *paragallinarum* (S3 Table). Altogether, these results suggest that *S*. *chromogenes* probably facilitates *Av*. *paragallinarum* infection in the respiratory tract due to the high isolation rate, colocalization rate and positive satellitism phenotype.

To dissect the interaction between *Av*. *paragallinarum* and *S*. *chromogenes*, we characterized the virulence genes of both *Av*. *paragallinarum* and *S*. *chromogenes* based on whole-genome sequencing. Genes encoding type IV pili (*piliA*, *piliB* and *piliQ*) and exopolysaccharides (*pgi*, *manA*, *manB*, *mrsA*, *galE* and *galU*) were detected in *Av*. *paragallinarum* compared to other *Haemophilus* isolates (S5A Fig). Moreover, we detected the presence of genes responsible for cell adhesion and immune evasion in these *S*. *chromogenes* isolates, including *atl*, *capO*, *capD*, *adsA* and *sbi*. Notably, genes encoding toxins, such as beta hemolysin (*hlb*), lipases (*lip* and *geh*) and thermonuclease (*nuc*), were also confirmed (S5B Fig), which have been identified in *S*. *chromogenes*-mediated mastitis of bovines and goats [29, 30]. Taken together, our results indicate that both *S*. *chromogenes* and *Av*. *paragallinarum* isolated from the respiratory tract of birds are likely opportunistic pathogens with the potential to express diverse virulence factors.

### *S*. *chromogenes* directly facilitated the survival of *Haemophilus in vitro*

**Fig 2:**
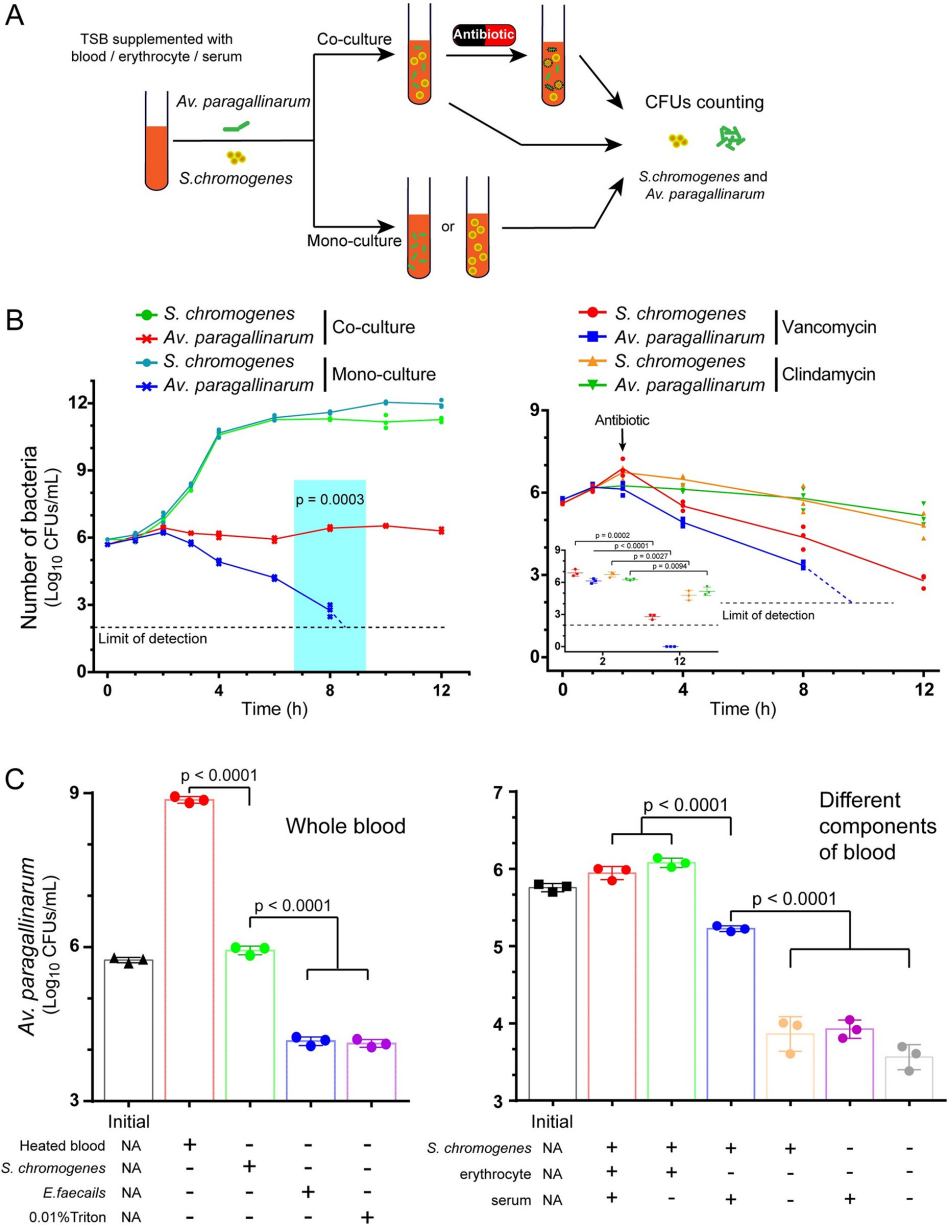
*S*. *chromogenes* supports the survival of *Av*. *paragallinarum in vitro*. (A) Workflow of *in vitro* coculture experiments. *S*. *chromogenes* SC10 and *Av*. *paragallinarum* X1-1S-1 were cocultured or monocultured in homemade broth media with or without antimicrobial agents. The CFUs/mL bacteria of all groups were subsequently calculated. (B) The growth dynamic of *S*. *chromogenes* and *Av*. *paragallinarum* in the presence of 5% (v/v) whole blood with (picture on the right) or without antimicrobial agents (picture on the left). LOD, limit of detection. *P* values were determined by unpaired *t*-test. The values of three biological replicates are shown as individual points (n = 3). (C) Replication of *Av*. *paragallinarum* in the presence of whole blood or blood components. NA, not applicable. *P* values were determined by one-way ANOVA. The mean of three biological replicates is shown, and error bars represent the standard deviation (SD) (n  =  3).

To estimate the connection between *S*. *chromogenes* and *Av*. *paragallinarum*, we first examined their growth rate using homemade broth media supplemented with different blood components (Fig 2A). We observed that *Av*. *paragallinarum* died gradually in the medium, and no living cells were detected after monoculture for 10 h. Conversely, both *Av*. *paragallinarum* and *S*. *chromogenes* survived in the coculture system (Fig 2B). To obtain a better understanding of the role of *S*. *chromogenes*, we explored antibiotics (vancomycin and clindamycin) that specifically target Gram-positive bacteria in the coculture system. The number of *Av*. *paragallinarum* decreased rapidly in the presence of bactericidal vancomycin but remained constant under the treatment with bacteriostatic clindamycin (Figs 2B and S6A). These results imply that live *S*. *chromogenes* plays a crucial role in the maintenance and survival of *Av*. *paragallinarum*. Additionally, we tested other bacteria, such as *Enterococcus faecalis*, which also exhibited high rates of isolation and colocalization; however, we found no such potentiation to *Av*. *paragallinarum* (Fig 2C). Therefore, it seems that there is a unique interaction between *Av*. *paragallinarum* and *S*. *chromogenes*.

**Fig 3:**
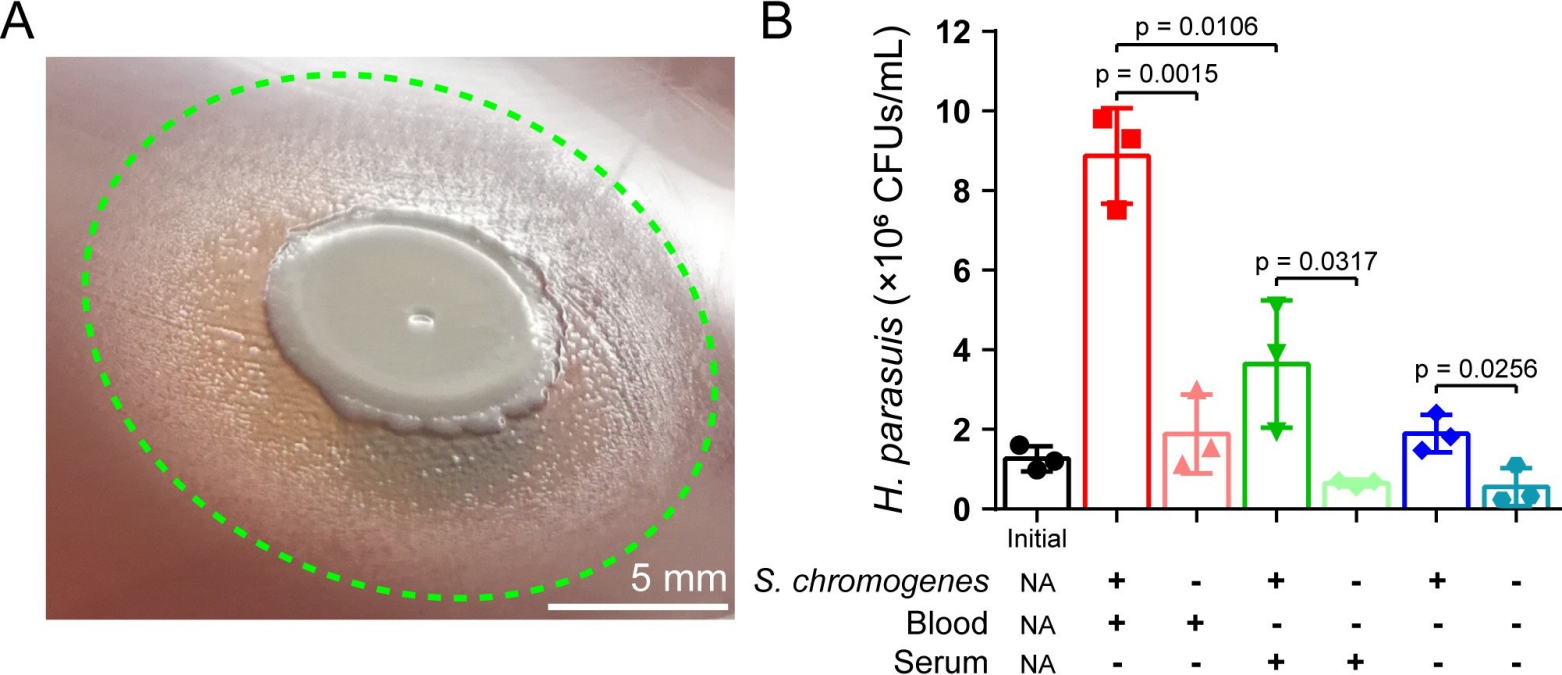
*S*. *chromogenes* supports the growth of *H*. *parasuis in vitro*. (A) Satellitism formed by *S*. *chromogenes* and *H*. *parasuis* after coculturing for 48 h. (B) *H*. *parasuis* displayed high density in the presence of *S*. *chromogenes*, together with either blood or serum. *H*. *parasuis* was monocultured or cocultured with *S*. *chromogenes* in TSB supplemented with different nutrients for 6 h. NA, not applicable. *P* values were determined by unpaired *t*-test. The mean of three biological replicates is shown, and error bars represent the standard deviation (SD) (n  =  3).

Interestingly, *Av*. *paragallinarum* grew well in tryptic soy broth (TSB) supplemented with heat-treated blood. Heating blood can not only inactivate NADase but also promote the release of intracellular NAD^+^ to facilitate the growth of *Haemophilus* [31]. In contrast, blood cell lysis by Triton cannot support bacterial replication, although such surfactant is nontoxic to *Av*. *paragallinarum* (Figs 2C and S6A). To further understand the effect of *S*. *chromogenes*, we examined the survival of *Av*. *paragallinarum* in media supplemented with different blood components. Remarkably, *Av*. *paragallinarum* grew to high density in the presence of *S*. *chromogenes* together with either whole blood or erythrocytes (Fig 2C). These results suggest that the presence of *S*. *chromogenes* is a prerequisite for the survival of *Av*. *paragallinarum*. To extend our observation, we verified the interaction between *S*. *chromogenes* and *Haemophilus parasuis*, which typically causes Glässer’s disease in pigs [32]. We confirmed that *H*. *parasuis* could not grow on blood agar in the absence of *S*. *chromogenes* (Fig 3A). Additionally, *S*. *chromogenes* significantly increased the number of *H*. *parasuis* in the medium supplemented with blood or serum (Fig 3B). Therefore, the promotional effect of *S*. *chromogenes* on the growth of *Haemophilus* spp. should be universal.

**Fig 4:**
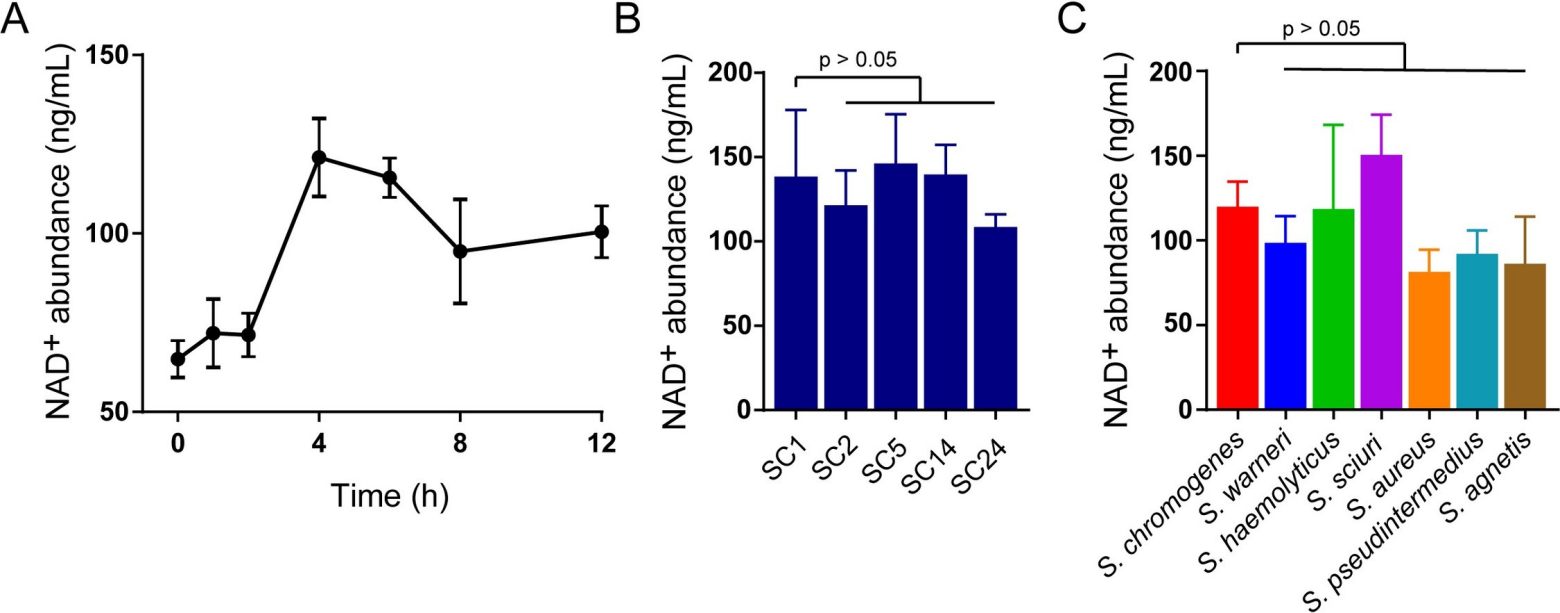
Accumulation of *Staphylococcus*-derived NAD^+^ promotes the survival of *Av*. *paragallinarum*. (A) The NAD^+^ content in the medium. *S*. *chromogenes* SC10 was monocultured in TSB supplemented with 5% (v/v) serum at 37°C. (B) NAD^+^ content in cultures of five *S*. *chromogenes* isolates after 4 h. (C) NAD^+^ content in cultures of different *Staphylococcus* isolates after 4 h at 37°C. *P* values were determined by one-way ANOVA. The mean of three biological replicates is shown, and error bars represent the standard deviation (SD) (n  =  3).

To investigate the underlying mechanism, we directly measured the NAD^+^ content in the culture medium using a colorimetric assay [33]. The accumulation of NAD^+^ reached its peak after culturing *S*. *chromogenes* for 4 h (Fig 4A). Interestingly, we found that the NAD^+^ content in the supernatants of cultures of various *S*. *chromogenes* isolates exhibited similar levels (Fig 4B). Similarly, other species of *Staphylococcus* showed the capability to provide NAD^+^ (Fig 4C). We observed that both the NAD^+^ content and bacterial number increased when *S*. *chromogenes* was cultured at 42°C (Figs 4C and S7A and S7B), implying that *S*. *chromogenes* probably adapts to the body temperature of birds. Although the sole *Staphylococcus sciuri* isolate displayed high NAD^+^ production, there was no difference in the growth of *Av*. *paragallinarum* when cocultured with either *S*. *chromogenes* or *S*. *sciuri* (S7C and S7D Fig). Collectively, our findings indicate that the prevailing *S*. *chromogenes* may dominantly provide sufficient NAD^+^ for the survival of *Av*. *paragallinarum*.

### *S*. *chromogenes* exacerbated the infection of *Av*. *paragallinarum*

**Fig 5:**
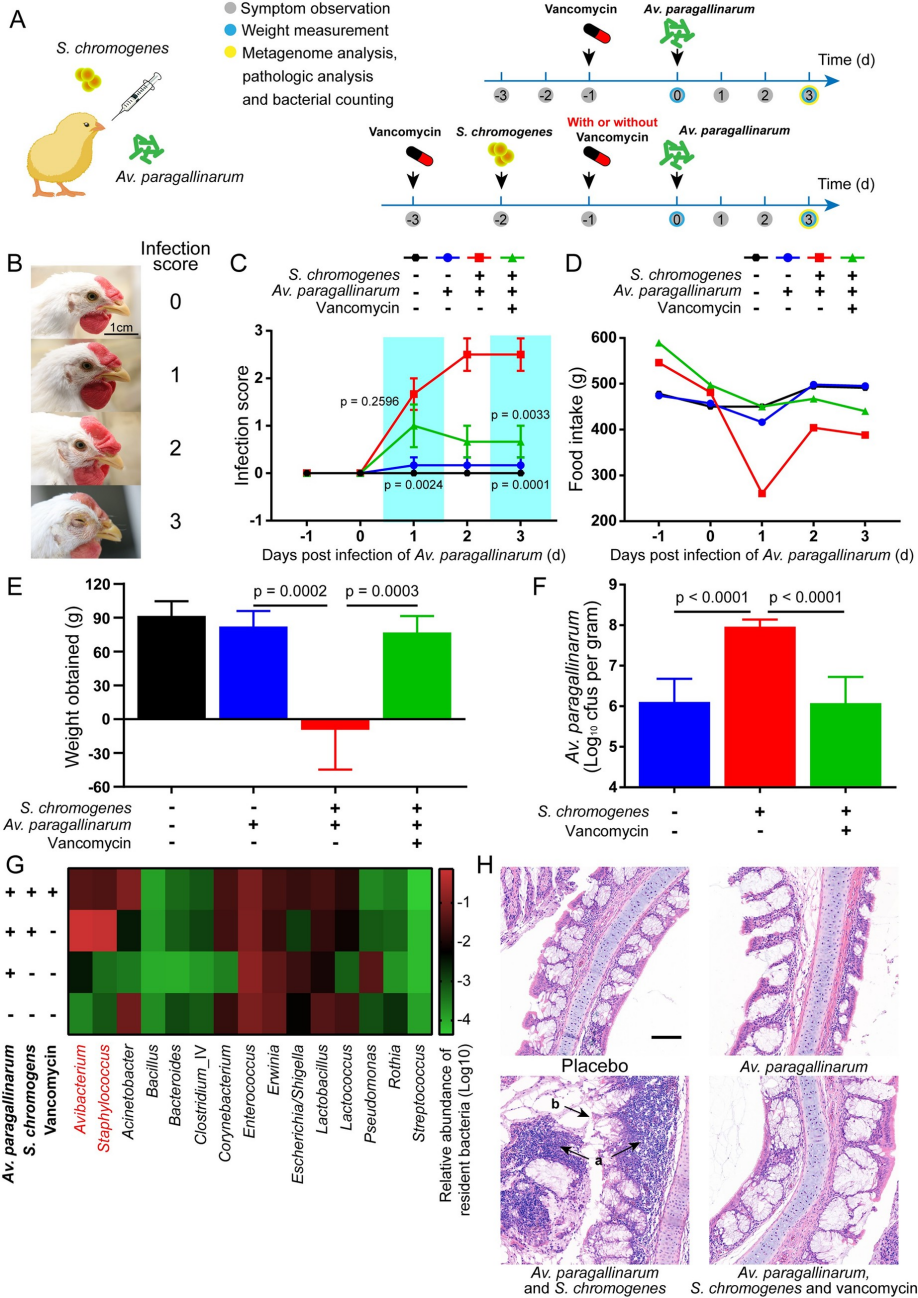
*S*. *chromogenes* aggravates the infection of *Av*. *paragallinarum*. (A) Scheme of animal models. SPF white leghorn chickens were randomly divided into four groups with six chickens in each group. Bacterial infection (10^9^ CFUs per bird), antimicrobial treatment, evaluation of food and water intake, and observation of clinical symptoms were all completed in the morning of each day. (B) Infection scores of symptoms. (C) *S*. *chromogenes* aggravated the clinical symptoms of birds infected with *Av*. *paragallinarum*. The difference was compared among the vancomycin treatment group and mono- or coinfection groups. (D) The food intake of each group was recorded every morning before renewal. (E) Body weight was recorded pre- and post-trial, and the weight obtained was calculated using subtraction. (F) Bacterial burden of the nasal cavity and infraorbital sinuses. (G) The bacterial community of the nasal cavity and infraorbital sinuses. (H) Serious tissue damage was observed in birds coinfected with *S*. *chromogenes* and *Av*. *paragallinarum*. A large number of inflammatory cells infiltrated the epithelial tissue or were contained in the respiratory secretions (a). Epithelial cells were damaged and shed (b). Scale bar = 100 μm. *P* values were determined by unpaired *t*-test. The mean of six biological replicates is shown, and error bars represent the standard deviation (SD) (n  =  6).

Given that *S*. *chromogenes* promotes the survival of *Av*. *paragallinarum in vitro*, we next investigated the role of *S*. *chromogenes* in animal models. Briefly, six-week SPF birds were randomly divided into four groups with six birds in each group and challenged with *Av*. *paragallinarum* alone or accompanied by *S*. *chromogenes* sequentially. Moreover, vancomycin was applied not only to remove the intrinsically colonized *Staphylococcus* spp. but also to kill *S*. *chromogenes* after infection (Fig 5A). The birds coinfected with *Av*. *paragallinarum* and *S*. *chromogenes* showed more severe clinical symptoms than those infected with *Av*. *paragallinarum* alone (Figs 5B–5E and S8). Consistently, the burden of *Av*. *paragallinarum* in the nasal cavity and infraorbital sinuses increased approximately 100 times compared with the monoinfection (Fig 5F and 5G). Although the relative abundances of *Erwinia*, *Lactobacillus* and *Pseudomonas* increased (Fig 5G), none of them were isolated clinically (S3 Table). Remarkably, we observed extensive infiltration of inflammatory cells and detachment of epithelial cells in the coinfected group (Fig 5H), which are in agreement with previous reports that coinfection of *Av*. *paragallinarum* and other bacteria exacerbates the symptoms in birds [34, 35]. As expected, the removal of *S*. *chromogenes* by vancomycin significantly prevented the progression of disease caused by *Av*. *paragallinarum* and decreased the bacterial burden (Figs 5C–5H and S8). However, the burden of *Av*. *paragallinarum* in the tissue still reached approximately 10^6^ CFUs/g in the absence of *S*. *chromogenes*. We deduced that the propagation of *Av*. *paragallinarum* may be directly promoted by the host because host cells might release NAD^+^ into the extracellular environment under conditions of inflammation or cellular stresses [14, 36]. Hence, our data indicate that resident *S*. *chromogenes* in the upper respiratory tract greatly promotes *Av*. *paragallinarum* infection in birds.

### *S*. *chromogenes* and *Av*. *paragallinarum* promoted the synthesis and release of NAD^+^

**Fig 6:**
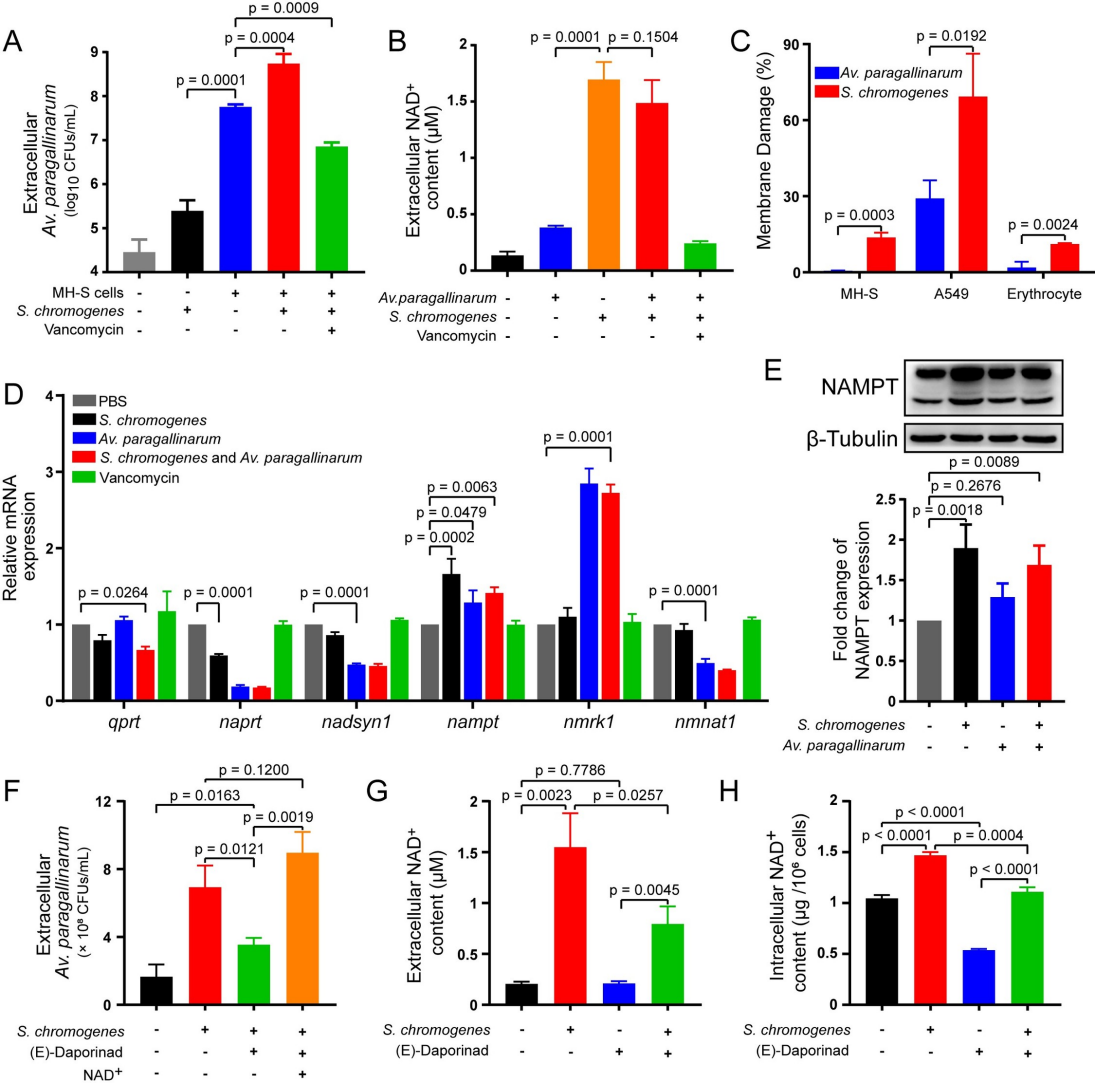
*S*. *chromogenes* hijacks host cells to promote the growth of *Av*. *paragallinarum*. (A) *S*. *chromogenes* increased the number of extracellular *Av*. *paragallinarum*. MH-S cells were infected with *S*. *chromogenes* SC10 and *Av*. *paragallinarum* X1-1S-1 (MOI = 1) and treated with 10 μg/mL vancomycin for 6 h. (B) *S*. *chromogenes* promoted NAD^+^ release from the host cells. MH-S cells were treated the same as in (A). (C) *S*. *chromogenes* seriously damaged the membrane of mammalian cells. MH-S and A549 cells were infected with *S*. *chromogenes* SC10 (MOI = 1) for 6 h, and erythrocytes were coincubated with *S*. *chromogenes* (10^6^ CFUs/mL) in PBS for 6 h. (D) The mRNA expression of NAD^+^ synthetases. MH-S cells were infected with *S*. *chromogenes* SC10 and/or *Av*. *paragallinarum* X1-1S-1 (MOI = 1) or incubated with 10 μg/mL vancomycin for 6 h. (E) *S*. *chromogenes* promoted the expression of NAMPT. A549 cells were infected with *S*. *chromogenes* SC10 and/or *Av*. *paragallinarum* X1-1S-1 (MOI = 1) for 6 h. (F) The NAMPT inhibitor (E)-daporinad reduced the number of *Av*. *paragallinarum*. MH-S cells were infected with *S*. *chromogenes* SC10 and *Av*. *paragallinarum* X1-1S-1 (MOI = 1) and treated with 1 μM (E)-daporinad and/or 10 μg/mL exogenous NAD^+^ for 6 h. (G, H) *S*. *chromogenes* increased the content of extracellular or intracellular NAD^+^. MH-S cells were infected with *S*. *chromogenes* (MOI = 1) and/or treated with 1 μM (E)-daporinad for 6 h. *P* values were determined by one-way ANOVA (A, B, D and E) and unpaired *t*-test (C, F, G and H). The mean of three biological replicates is shown, and error bars represent the standard deviation (SD) (n  =  3).

To track the source of NAD^+^ derived from *S*. *chromogenes* and/or host cells, we employed a pulmonary epithelial cell line (A549) and macrophage cell line (MH-S) to explore the intrinsic relationship between hosts and bacteria. No propagation of *Av*. *paragallinarum* was observed in the absence of host cells (Figs 6A and S10A). Nevertheless, the number of *Av*. *paragallinarum* significantly increased and reached approximately 100-fold higher than the initial bacterial density in the presence of MH-S cells or A549 cells after incubation for 6 h. Comparably, the number of *Av*. *paragallinarum* increased approximately another 10-fold in the presence of *S*. *chromogenes* (Figs 6A and S10A and S10B). Subsequently, we quantified the NAD^+^ content in the supernatant of the cell cultures. In contrast to the threefold increase in extracellular NAD^+^ content with MH-S cells, the content of extracellular NAD^+^ increased approximately 10 times when cocultured with *S*. *chromogenes* (Fig 6B). Considering that *S*. *chromogenes* alone could not promote the growth of *Av*. *paragallinarum* (S10A Fig), the great accumulation of extracellular NAD^+^ was more likely to be released from host cells. Therefore, *S*. *chromogenes* should play a critical role in elevating the NAD^+^ level originating from host cells during coinfection.

**Fig 7:**
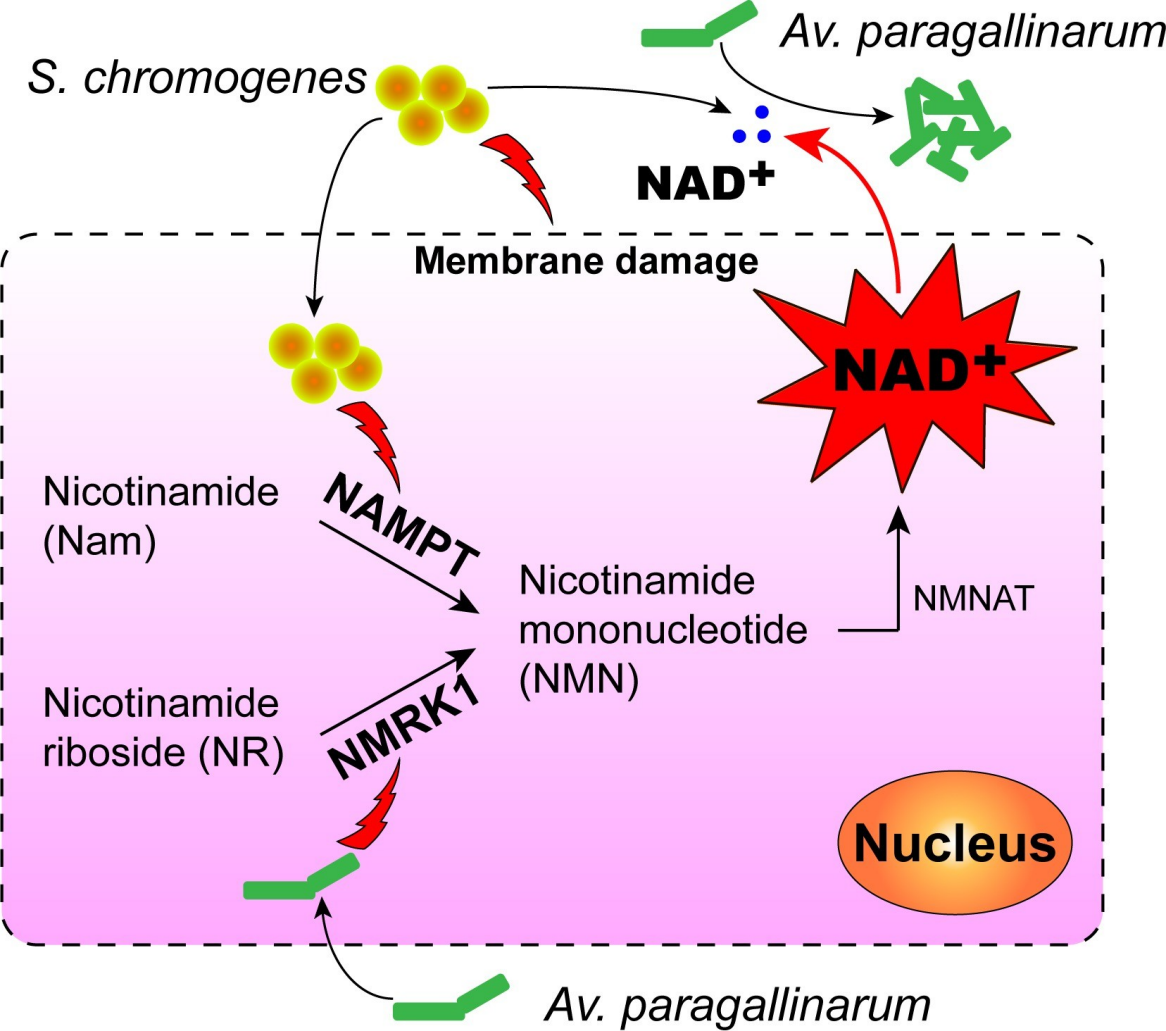
Resident bacteria promote the replication of *Av*. *paragallinarum*. Resident bacteria such as *S*. *chromogenes* enhance NAD^+^ biosynthesis and release by host cells in the respiratory tract. Consequently, the increased NAD^+^ level in the extracellular environment accelerates the replication of *Av*. *paragallinarum*.

To further investigate the release of NAD^+^ from host cells, we observed that *S*. *chromogenes* caused more severe membrane damage to all mammalian cells, particularly epithelial cells (Fig 6C), than *Av*. *paragallinarum*. Because NAD^+^ can be released from lysed cells into the extracellular environment [14], our results indicated that NAD^+^ may be passively released from host cells due to membrane damage. Interestingly, we found that the synthesis of NAD^+^ in host cells was activated by either *S*. *chromogenes* or *Av*. *paragallinarum* (Figs 6D, 6E and S10F and S13A). It seems that both bacteria tend to promote the production of NMN in host cells, a crucial intermediate for NAD^+^ biosynthesis [37], through the upregulation of two different pathways (Fig 7). Notably, the inhibition at the transcriptional level caused by *Av*. *paragallinarum* may not immediately reduce the amount of NMNAT1 (catalyzes the formation of NAD^+^ from NMN) and the downstream product NAD^+^ in the short term (S13B Fig). However, *Av*. *paragallinarum* may interrupt the homeostasis of intracellular NAD^+^ and induce the apoptosis of immune cells with persistent inhibition [38] to promote invasion.

Compared to other *Staphylococcus* isolates, such as *Staphylococcus warneri* and *S*. *aureus*, *S*. *chromogenes* showed a better growth-promoting effect on *Av*. *paragallinarum* by enhancing the expression of *nampt* (S12 Fig). Subsequently, we employed a specific inhibitor of NAMPT, (E)-daporinad [39], to test the role of NAMPT in the growth of *Av*. *paragallinarum*. (E)-Daporinad sharply reduced the number of extracellular *Av*. *paragallinarum*. Nevertheless, exogenous addition of NAD^+^ completely abolished this inhibitory effect (Figs 6F and S6B), as confirmed by the diminished extracellular and intracellular NAD^+^ contents (Fig 6G–6H). Therefore, these results highlight that NAD^+^ production in host cells is hijacked by *S*. *chromogenes* to promote the growth of *Av*. *paragallinarum*. Moreover, we observed the presence and survival of internalized *S*. *chromogenes* or *Av*. *paragallinarum* in epithelial cells and macrophages based on confocal microscopy analysis and gentamycin protection assays (S14A and S14B Fig), consistent with previous studies showing that *Haemophilus* spp. and other bacteria can also initiate intracellular survival [40–42]. Hence, we deduce that *S*. *chromogenes* not only promotes the growth of *Av*. *paragallinarum* under extracellular conditions but also increases the intracellular burden of *Av*. *paragallinarum* (S14C and S14E Fig), through either directly facilitating the invasion of extracellular *Av*. *paragallinarum* or promoting the growth of internalized *Av*. *paragallinarum*. Altogether, our results indicate that *S*. *chromogenes* not only destabilizes the membrane of host cells but also induces the biosynthesis of NAD^+^ to consequently promote *Av*. *paragallinarum* growth (Fig 7).

## Discussion

Our results highlight the contribution of resident bacteria to *Av*. *paragallinarum* infection in the respiratory tract and most likely the infection of other *Haemophilus* spp.. Resident bacteria such as *S*. *chromogenes* can not only directly supply NAD^+^ to *Av*. *paragallinarum* but also reinforce the biosynthesis and release of NAD^+^ in host cells. Hence, *Av*. *paragallinarum* succeeded in hijacking host cells to become its own NAD^+^ sources (Fig 7). Our findings elucidate a better understanding of the infection caused by opportunistic pathogens in the respiratory tract and the development of new strategies to prevent and control such infections.

Commensal *Haemophilus* spp. are opportunistic pathogens that cause volatile infections [43]. Most *Haemophilus* need NAD^+^ or NMN as an extra growth factor, whereas the level of such compounds usually cannot reach satisfactory concentrations for rapid bacterial replication in the respiratory tract of healthy individuals [10–12, 44]. Unexpectedly, we found that resident *S*. *chromogenes* along with other *Staphylococcus* isolates can produce additional NAD^+^ (Figs 4A–4C and S7) to promote the survival of *Av*. *paragallinarum*. Consistent with our findings (S12 Fig), previous studies also suggest that the colonization of *H*. *influenzae* increases in hosts precolonized with either *S*. *aureus* or *Streptococcus pneumoniae*, which are resident bacteria in the upper respiratory tract [45, 46]. Mammalian cells have evolved three ways to produce NAD^+^. Coincidently, both *S*. *chromogenes* and *Av*. *paragallinarum* activate the salvage pathway (Figs 6D and S10F), of which either the intermediate (NMN) or end-product (NAD^+^) can promote the growth of *Av*. *paragallinarum* [44]. This hijacking behavior suggests a novel synergistic infection in which opportunistic pathogens maximally plunder resources from host cells with the assistance of resident bacteria.

Interestingly, we found that *S*. *chromogenes* adapted better to high culturing temperatures at 42°C than at 37°C (Figs 3B and S7B). Previous reports indicate that tolerance to high temperatures is highly related to bacterial virulence for better investments in resource acquisition and other purposes [47, 48]. In contrast to those in bovine-originating *S*. *chromogenes* [29, 49], the diversity of virulence genes in bird-derived isolates is much lower (S5 Fig). The compromise of the survival of *S*. *chromogenes* may attenuate its pathogenicity to hosts, which is consistent with our observation that clinical symptoms were barely recognized after birds were infected solely with *S*. *chromogenes* (S9 Fig). Notably, temperature adaptability may explain its irreplaceable role in infections of the avian respiratory tract due to intrinsically high body temperatures ranging from 40 to 43°C.

Although we have demonstrated that resident bacteria make great contributions to infections, how *Av*. *paragallinarum* evades innate immune killing and the restriction of local microbial communities to acquire a replication niche remain unclear. Moreover, vaccination is the most important strategy against IC, and the combined immunization of *S*. *chromogenes* and *Av*. *paragallinarum* to produce better outcomes needs further evaluation.

## Materials and methods

### Epidemiological investigation

We undertook a study to investigate the prevalence of infectious coryza (IC) in 2019. Natural and anthropogenic data from 14 provinces and municipalities in China were collected by questionnaires shared on the internet. Multiple anthropogenic and natural factors were included. Statistical analyses were based on previously described methods [50]. Variables with *P* < 0.05 were kept in the final model.

### Bacterial isolation and culture

Twenty birds with typical facial edema and discharges in the nasal cavity and infraorbital sinuses were sacrificed for bacterial isolation. The heads of birds were covered with 75% ethyl alcohol to avoid contamination. Samples were collected from the nasal cavity and infraorbital sinuses when beaks were cut and jowls were slit after the volatilization of ethyl alcohol. The discovery method was culture-based isolation. In detail, the initial inoculations were performed on blood agar plates, and tryptic soy agar plates (TSA, Beijing Land Bridge Technology Co., China) supplemented with 5% (v/v) fetal bovine serum (FBS, Gibco, Thermo Fisher Scientific, US) and 0.001% (w/v) nicotinamide adenine dinucleotide (NAD^+^, Sigma-Aldrich, Merck Millipore, US) were used at the same time. Samples were then incubated in a 5% CO_2_ incubator at 37°C for 48 h. The suspected colonies of *Av*. *paragallinarum* were then cultured in tryptic soy broth (TSB, Beijing Land Bridge Technology Co., China) supplemented with 5% (v/v) FBS and 0.0025% (w/v) NAD^+^. Other colonies were cultured in TSB for further identification.

### Antibacterial tests

The minimal inhibitory concentrations (MICs) of different antibiotics for bacteria were determined using the broth microdilution method according to the Clinical and Laboratory Standards Institute criteria described in documents VET08 and M100-S28 [51, 52]. Briefly, antibiotics were diluted twofold in Mueller–Hinton broth (MHB, Beijing Land Bridge Technology Co., China) and mixed with an equal volume of bacterial suspensions in MHB containing approximately 1.5×10^6^ colony-forming units (CFUs)/mL in a clear, UV-sterilized, 96-well microtiter plate (Corning Incorporated Co., US). After *S*. *chromogenes* was cultured for 18 h at 37°C, the MICs were defined as the lowest concentrations of antibiotics with no visible growth of bacteria. For *Av*. *paragallinarum* isolates, the only modification was the broth used: cation-adjusted Mueller-Hinton broth (CAMHB, Shanghai GuanDao Biotech Co., China) plus 0.0025% (w/v) NAD^+^ and 1% (v/v) sterile-filtered heat-inactivated chicken serum (Solarbio Life Science Co., China). Additionally, the standard quality control strains for MIC tests were *S*. *aureus* American Type Culture Collection (ATCC) 29213 and *E*. *coli* ATCC 25922.

### Bacterial identification and serotyping of *Av*. *paragallinarum* isolates

The identification of bacterial species was conducted by matrix-assisted laser desorption/ionization time-of-flight (MALDI-TOF) mass spectrometry analysis and 16S rRNA gene sequencing. DNA was extracted from all isolates using a TIANamp Bacteria DNA Kit (Tiangen Biotech Co., Beijing, China) according to the manufacturer’s instructions. *Av*. *paragallinarum* isolates were serotyped using multiplex polymerase chain reaction (mPCR) to identify serogroups (A, B, and C), as previously described [25]. The isolation rates and colocalization rates were calculated by the following formulae.

Isolation rate: the number of bacteria of a specific genus/total number of samples.

Colocalization rate: the number of birds infected with a specific genus of bacteria and *Av*. *paragallinarum*/the number of birds infected with *Av*. *paragallinarum*.

### Satellitism

The workflow of these experiments referred to a previously described method, with some modifications [23]. The *Av*. *paragallinarum* isolates X1-1S-1 and 12-4N-1 and *Haemophilus parasuis* 17CQ1B34-2 [32] were selected for the tests. Briefly, 100 μL of phosphate-buffered saline (PBS, pH = 7.4) suspension of *Av*. *paragallinarum* (10^6^ CFUs/mL) prepared from 24-hour growth on a TSA plate supplemented with FBS and NAD^+^ was plated on a sheep blood agar plate. The overnight growth of bacteria of other genera was adjusted to McFarland 0.5 turbidity in 0.9% NaCl solution, and 2 μL of suspension was subsequently inoculated onto the blood agar plates, which were covered with *Av*. *paragallinarum* or *H*. *parasuis*. When the droplets were dry, the plates were incubated in a 5% CO_2_ incubator at 37°C. The radii of satellitism were measured after incubation for 48 h. *S*. *aureus* ATCC 29213 and methicillin-resistant T144 [53] were set as reference strains.

### Growth curves and bacterial time-killing curves

The workflow of these experiments referred to a previously described method, with some modifications [54]. Briefly, overnight cultures of the *S*. *chromogenes* isolate SC10, *Staphylococcus agnetis* isolate 12-4S-3, *S. aureus* isolate 12-1N-1, *Staphylococcus haemolyticus* isolate X2-2N-1, *Staphylococcus warneri* isolate X1-2S-2, *Staphylococcus pseudintermedius* isolate 29HD2S-5 and *Staphylococcus sciuri* isolate Z1S-3 were adjusted to McFarland 0.5 turbidity and diluted 1:100 in TSB medium. The overnight culture of the *Av*. *paragallinarum* isolate X1-1S-1 was adjusted to McFarland 0.5 turbidity and diluted 1:100 in TSB medium supplemented with 5% (v/v) FBS and 0.0025% (w/v) NAD^+^. Alternatively, 0.02% Triton, 16 μg/mL clindamycin, 64 μg/mL vancomycin, or 2 μM (E)-daporinad (MedChemExpress Co., US) was added to a 96-well microplate and mixed with an equal volume of bacterial culture with the plate lid covering. The overnight culture of the *S*. *chromogenes* isolate SC10 was adjusted to McFarland 0.5 turbidity and diluted 1:100 in TSB medium supplemented with 5% (v/v) FBS. (E)-Daporinad (2 μM) was added to a 96-well microplate and mixed with an equal volume of bacterial culture with the plate lid covering. The growth curves were recorded afterward at a wavelength of 600 nm, with an interval of 1 h at 37°C or 42°C, using an Infinite M200 Microplate reader (Tecan Co., Switzerland) in the long-term model.

The workflow of bacterial time-killing curve experiments was performed according to a previously described method, with some modifications [53]. The overnight cultures of the *S*. *chromogenes* isolate SC10 and *Av*. *paragallinarum* isolate X1-1S-1 were adjusted to McFarland 0.5 turbidity and diluted 1:100 in TSB medium supplemented with 5% (v/v) sheep blood. In addition, vancomycin and clindamycin (Aladdin Biochemical Technology Co., Shanghai, China) were applied at 32 μg/mL and 8 μg/mL, respectively. All media were cultured in 12 mL sterile culture tubes (Haimen United Laboratory Equipment Development Co., Jiangsu, China), the caps of which were equipped with filters so the gas could be normally exchanged. The suspension of all experiments was serially diluted tenfold and plated on solid medium, with incubation at 37°C for 24 h. TSA plates were applied for the detection of *S*. *chromogenes*, while TSA plates supplemented with 5% (v/v) FBS, 0.001% (w/v) NAD^+^ and 10 μg/mL vancomycin were used for the examination of *Av*. *paragallinarum*.

### Coculture of *S*. *chromogenes* and *Av*. *paragallinarum in vitro*

Overnight cultures of the *S*. *chromogenes* isolate SC10, *Av*. *paragallinarum* isolate X1-1S-1, *Enterococcus faecali*s isolate 29HD2S-2 (from this research), *S*. *sciuri* isolate Z1S-3, and *H*. *parasuis* isolate 17CQ1B34-2 were first adjusted to McFarland 0.5 turbidity in TSB. Blood was heated at 85°C for 10 min. Erythrocytes and serum were separated by centrifugation of fresh blood at 500 × g for 10 min, and erythrocytes were resuspended in isovolumetric 0.9% NaCl solution. Bacterial suspensions were diluted 1:100 in TSB medium supplemented with 5% (v/v) heated blood, fresh blood, fresh blood with 0.01% (v/v) Triton, resuspended erythrocytes or isolated serum separately or together. The numbers of *Av*. *paragallinarum* in the medium were detected by plating serial dilutions on TSA supplemented with 5% (v/v) FBS and 0.0025% (w/v) NAD^+^ after bacterial culture for 6 h at 37°C.

### Whole genome sequencing analysis

DNA extraction, library construction, sequencing, assembly, and construction of SNP-based phylogenetic trees were completed according to a previous report [55]. Antibiotic resistance genes were identified using BLAST in a custom database, and virulence genes of *Av*. *paragallinarum* and *S*. *chromogenes* isolates were identified using BLAST by a custom database retrieved from VFDB (http://www.mgc.ac.cn/cgi-bin/VFs/compvfs.cgi?Genus=Haemophilus, http://www.mgc.ac.cn/cgi-bin/VFs/compvfs.cgi?Genus=Staphylococcus). The whole-genome sequences of *Av*. *paragallinarum* and *S*. *chromogenes* isolates investigated in this study have been deposited at GenBank under accession numbers PRJNA648655 and PRJNA648661.

### Ethics statement

All animal protocols were approved by the Genentech Institutional Animal Care and Use Committee at China Agricultural University (SYXK, 2018–0038). The experimental procedures involving birds gained approval (AW40300202-2).

### Birds and animal regression test

Male specific pathogen-free (SPF) white leghorn chickens aged five weeks were obtained from Beijing Boehringer Ingelheim Vital Biotechnology Co., Ltd. Birds were randomly divided into four groups with six birds in each group and adapted to standardized environmental conditions under negative pressure for one week before infection. Overnight cultures of the *S*. *chromogenes* isolate SC10 and *Av*. *paragallinarum* isolate X1-1S-1 were resuspended in 200 μL of 0.9% saline solution at 1 × 10^9^ CFUs per bird, and vancomycin was prepared at a concentration of 32 μg/mL. Vancomycin treatment and bacterial infection were both applied by injection in the infraorbital sinuses, with a volume of 200 μL. On the last day of the experiments, all birds were sacrificed. The tissues of the nasal cavity and infraorbital sinuses were removed and homogenized in sterile PBS for the determination of bacterial load and metagenome analysis, or tissue was removed and fixed in 4% paraformaldehyde for hematoxylin and eosin (H & E) staining.

### Clinical scoring

Birds were examined daily, and clinical signs were scored and recorded every morning throughout the whole experiment according to a previously reported scale [35]: 0, no clinical signs; 1, mild nasal discharge or swelling of the infraorbital sinuses; 2, nasal discharge along with swelling of the infraorbital sinuses; and 3, severe swelling of the infraorbital sinuses with or without conjunctivitis. The body weights of birds were recorded pre- and post-trial. The food and water intakes of each group of birds were measured and recorded every morning before renewal.

### Bacterial community characterization

Bacterial community characterization was performed according to previously described research [56]. Briefly, tissue samples of the nasal cavity and infraorbital sinuses were collected and homogenized in sterile PBS. The V3-V4 regions of microbial 16S rDNA genes were amplified and purified, and sequencing was performed at an institute authorized to sequence environmental genomics (Sinobiocore Inc., Beijing, China). Raw fastq files were demultiplexed using barcode sequences (https://github.com/jameslz/div-utils/blob/master/div-utils version: 0.0.1-r1-dirty). Quality filtering was performed using Trimmomatic [57], and paired reads were merged using the USEARCH fastq_mergepairs command [58]. Operational taxonomic units (OTUs) were clustered with a 97% similarity cutoff using USEARCH UPARSE [59], and the chimeric sequences were removed in the UPARSE pipeline. The raw sequence reads have been deposited in the NCBI Sequence Read Archive under accession number PRJNA646660.

### Cell lines and cell culture

Human lung carcinoma epithelial cells (A549) and mouse lung macrophages (MH-S) were cultured in Dulbecco’s modified Eagle’s medium (DMEM, Thermo Fisher Scientific, US) or Roswell Park Memorial Institute (RPMI)-1640 medium (Thermo Fisher Scientific, US) supplemented with 10% FBS and 1% (w/v) penicillin-streptomycin (Solarbio Life Science Co., Shanghai, China). All cells were cultured at 37°C in a 5% CO_2_ atmosphere. All cell lines were obtained from the ATCC.

### Cell infection

A549 and MH-S cells were seeded in antibiotic-free medium at a concentration of 0.5 × 10^6^ cells per well in 24-well plates (Corning Incorporated Co., US). Cells were infected with the *S*. *chromogenes* isolate SC10, *S*. *aureus* isolate 12-1N-1, *S*. *warneri* isolate X1-2S-2, *Av*. *paragallinarum* isolate X1-1S-1, or *E*. *faecali*s isolate 29HD2S-2 (from this research) at a multiplicity of infection (MOI) of 1 and treated with 32 μg/mL vancomycin for 6 h. The numbers of *Av*. *paragallinarum* in the supernatant of cell cultures were subsequently detected by plating serial dilutions on TSA supplemented with 5% (v/v) FBS and 0.0025% (w/v) NAD^+^.

MH-S and A549 cells were seeded at 1 × 10^6^ cells per well in 12-well culture plates (Corning Incorporated Co., US) to form monolayers. Overnight cultures of the *S*. *chromogenes* isolate SC10 and *Av*. *paragallinarum* isolate X1-1S-1 were resuspended in PBS. Finally, bacterial resuspensions were diluted in DMEM or RPMI-1640 medium supplemented with 1% FBS and cocultured with A549 and MH-S cells at an MOI of 1 for 6 h. At the end of the trials, 100 μg/mL gentamycin was applied to remove the extracellular bacteria, and the counting of intracellular bacteria was followed by host cell lysis and serial dilution.

### Detection of membrane damage and cytotoxicity tests

Erythrocytes were harvested by centrifugation of fresh sheep blood at 500 × g for 10 min and subsequently resuspended in isovolumetric 0.9% NaCl solution. Overnight cultures of the *S*. *chromogenes* isolate SC10 or *Av*. *paragallinarum* isolate X1-1S-1 were standardized to match a 0.5 McFarland turbidity followed by a 1:100 dilution in PBS supplemented with a 10% (v/v) suspension of erythrocytes for 6 h at 37°C. After that, the fluids were first centrifuged at 500 × g for 10 min, and 200 μL of supernatants were centrifuged for another 2 min at 10000 × g. The membrane damage of erythrocytes was evaluated by detecting the released lactate dehydrogenase (LDH) in the supernatant.

A549 and MH-S cells were seeded in antibiotic-free medium at a concentration of 0.5 × 10^6^ cells per well in 24-well plates. Host cells were infected with *S*. *chromogenes* or *Av*. *paragallinarum* at an MOI of 1 or incubated with 0.01% Triton (Beyotime Biotechnology Co., Shanghai, China) as a positive control for 6 h. Supernatants of cell cultures were then acquired by centrifugation at 1000 × g for 5 min at 4°C. The levels of LDH in the supernatants were detected by an LDH cytotoxicity assay detection kit (Beyotime Biotechnology Co., Shanghai, China) following the manufacturer’s instructions.

Cytotoxicity examinations on mammalian cells were performed on A549 and MH-S cells using an LDH cytotoxicity assay detection kit following the manufacturer’s instructions. (E)-Daporinad (2–0.015625 μM) and 1×10^4^ cells were simultaneously added to 96-well plates. Cells were cultured in DMEM or RPMI-1640 medium supplemented with 1% heat-inactivated FBS and 1% (w/v) penicillin-streptomycin at 37°C in a 5% CO_2_ atmosphere for 12 h, followed by LDH tests.

### Detection of NAD^+^

Overnight cultures of different *Staphylococcus* isolates were standardized to match a 0.5 McFarland turbidity followed by a 1:100 dilution in TSB medium supplemented with 5% (v/v) FBS. The *S*. *chromogenes* isolate SC10 was cultured at 37°C for different times or at 42°C for 4 h. The *S*. *chromogenes* isolates SC1, SC2, SC5, SC14, and SC24 and other *Staphylococcus* isolates were cultured at 37°C for 4 h. Two hundred microliters of bacterial culture supernatant was obtained by centrifugation at 10000 × g for 2 min at 4°C.

MH-S and A549 cells were seeded in antibiotic-free medium at a concentration of 0.5 × 10^6^ cells per well in 24-well plates. Cells were then infected with the *S*. *chromogenes* isolate SC10 or *Av*. *paragallinarum* isolate X1-1S-1 at an MOI of 1 at 37°C for 6 h. In addition, infected MH-S cells were incubated with 32 μg/mL vancomycin or 1 μM (E)-daporinad at 37°C for 6 h. A549 cells were incubated with 0.01% Triton or 1 μg/mL LPS (Sigma-Aldrich, Merck Millipore, US) at 37°C for 6 h. The supernatants of cell cultures were acquired by centrifugation at 1000 × g for 5 min at 4°C. The extracellular or intracellular NAD^+^ levels were measured by an NAD^+^/NADH Assay Kit with WST-8 (Beyotime Biotechnology Co., Shanghai, China) following the manufacturer’s instructions.

### RT-qPCR analysis

RT-qPCR analyses were performed according to previously described research [60]. Briefly, MH-S and A549 cells were seeded in antibiotic-free medium at a concentration of 2 × 10^6^ cells per well in 6-well plates (Corning Incorporated Co., US). After that, the cells were infected with *S*. *chromogenes* SC10, SC1, SC2, SC5, SC14, or SC24; *Av*. *paragallinarum* X1-1S-1; *S*. *aureus* 12-1N-1; or *S*. *warneri* X1-2S-2 at an MOI of 1 or treated with 32 μg/mL vancomycin at 37°C for 6 h. Total RNA was extracted using a HiPure Total RNA Plus Mini Kit (Magen Biotechnology Co., Guangzhou, China), and 1 μg of extracted RNA was reverse transcribed with a PrimeScript RT Reagent Kit (TaKaRa Bio incorporated, Japan) following the manufacturer’s protocol. The messenger RNA levels of *qprt*, *naprt*, *nadsyn1*, *nampt*, *nmrk1* and *nmnat1* relative to those of the control genes *gapdh* or *actb* in MH-S or A549 cells were determined by real-time PCR tests with PowerUp SYBR Green Master Mix (Applied Biosystems, Thermo Fisher Scientific, US). RT-PCR tests were performed using the ABI Quantstudio 7 detection system (Applied Biosystems, Thermo Fisher Scientific, US). The fold changes in gene expression were determined using the 2^−ΔΔCt^ method. The primer sequences are listed in S6 Table.

### Western blotting

Western blotting was utilized to analyze the expression of NAMPT and NMRK1. A549 cells were seeded in antibiotic-free medium at a concentration of 2 × 10^6^ cells per well in 6-well plates. Cells were then infected with the *S*. *chromogenes* isolate SC10 and/or *Av*. *paragallinarum* isolate X1-1S-1 at an MOI of 1 at 37°C for 6 h. The primary antibodies included rabbit anti-NAMPT (Invitrogen, Thermo Fisher Scientific, US), rabbit anti-NRK1 (Abcam Co., UK) and rabbit anti-*β*-tubulin antibodies (Cell Signaling Technology, US). The secondary antibody was a goat anti-rabbit antibody (Beyotime Biotechnology Co., Shanghai, China). Gray values of protein bands were quantified by ImageJ software.

### Confocal laser scanning microscopy analysis

A549 cells were seeded in antibiotic-free medium at a concentration of 0.5 × 10^6^ cells per well in 24-well glass-bottom plates (NEST Life and Science Technology Co., Wuxi, China). Host cells were infected with pHrodoRed-labeled (Invitrogen, Thermo Fisher Scientific, US) *S*. *chromogenes* or *Av*. *paragallinarum* at an MOI of 20 for 1 h, followed by fixation in 4% paraformaldehyde. F-actin was stained with ActinGreen 488 ReadyProbes, and the nuclei were counterstained with DAPI (Beyotime Biotechnology Co., Shanghai, China).

For static images, fixed and stained cellular samples were captured by a Leica SP8 confocal microscope. To analyze the location of internalized bacteria, the Z-axis sections were cut every 0.3 μm. Images were analyzed and merged by LAS AF Lite software (Leica Biosystems, Germany).

### Statistical analysis

Statistical analysis was performed using GraphPad Prism 7.0 (GraphPad Software, Inc.). *p*-values were calculated using an unpaired *t*-test with Bonferroni correction between two groups or a nonparametric one-way ANOVA among multiple groups. All data are expressed as the mean ± SD. All experiments were performed on no less than three biological replicates.

## Supporting information

S1 Data Excel spreadsheet containing, in separate sheets, the underlying numerical data and statistical analysis for Figure panels 1D, 2B, 2C, 3B, 4A, 4B, 4C, 5C, 5D, 5E, 5F, 5G, 6A, 6B, 6C, 6D, 6E, 6F, 6G and 6H. (XLSX)

S2 Data Excel spreadsheet containing, in separate sheets, the underlying numerical data and statistical analysis for panels S3, S4, S6A, S6B, S7A, S7B, S7C, S7D, S8, S9, S10A, S10B, S10C, S10D, S10E, S10F, S11, S12, S13A, S13B, S14B, S14C, S14D and S14E Figs. (XLSX)

S1 Table Descriptive and univariable analysis of the variables of interest. *, Significant variable in univariable analysis (*P* ≤ 0.05). NA, not applicable. (XLSX)

S2 Table Descriptive and univariable analysis of factors associated with recurrent IC outbreaks. *, Significant variable in univariable analysis (*P* ≤ 0.05). NA, not applicable. (XLSX)

S3 Table Bacteria isolated from the respiratory tract of birds (excluding *Av*. *paragallinarum*). a, Colocalization rate: the number of birds infected with a specific genus of bacteria and *Av*. *paragallinarum*/the number of birds infected with *Av*. *paragallinarum*.
(XLSX)

S4 Table MICs of all tested antibiotics to *Av*. *paragallinarum*. FLO, florfenicol; CLI, clindamycin; ERY, erythromycin; CEF, cefoxitin; TET, tetracycline; OXA, oxacillin; ENR, enrofloxacin; TRI/SUL, trimethoprim/ sulfamethoxazole; GEN, gentamycin; PEN, penicillin; AMP, ampicillin; TIL, tilmicosin; AMO/CLA, amoxicillin/ clavulanic acid; MER, meropenem; POL, polymyxin E. a, Data represent the concentration of trimethoprim. b, Data represent the concentration of amoxicillin. c, The number of isolates with MIC values equal to or higher than the concentrations of tested ranges. d, The number of isolates with MIC values equal to or lower than the concentrations of tested ranges.
(XLSX)

S5 Table MICs of all tested antibiotics to *S*. *chromogenes*. MICs of antibiotics in μg/mL; PEN, penicillin; OXA, oxacillin; AMO/CLA, amoxicillin/clavulanic acid (2: 1); CEF, cefoxitin; ERY, erythromycin; TET, tetracycline; GEN, gentamycin; CLI, clindamycin; TIA, tiamulin; ENR, enrofloxacin; LIN, linezolid; RIF, rifampicin; VAN, vancomycin; TIL, tilmicosin; FLO, florfenicol; TRI/SUL, trimethoprim/sulfamethoxazole (1: 19).
(XLSX)

S6 Table RT-PCR primer sets in this study. *, Primers were obtained from PrimerBank (https://pga.mgh.harvard.edu/primerbank/index.html). References corresponding to primers: mice-*naprt* [61]; mice-*nampt* [62]; mice-*nmrk1* [63]; mice-*nmnat1*, mice-*gapdh* [64]; Human-*naprt*, Human-*nadsyn1*, Human-*nampt*, Human-*nmrk1* [60]; Human-*nmnat1* [65]; Human-*actb* [66].
(XLSX)

S1 Fig Mapped IC cases in China. Prevalence of IC in China: data from 103 poultry farms were collected by questionnaires shared on the internet. Map layers were obtained from Wikimedia Commons (https://commons.wikimedia.org/wiki/File:China-outline.svg).
(TIF)

S2 Fig Genetic Characteristics of *Av*. *paragallinarum* and *S*. *chromogenes* isolates. Core-genome SNP-based phylogenetic trees of *Av*. *paragallinarum* (A) and *S*. *chromogenes* (B). Bacteria of the two genera were divided into two genotypes (labeled with blue and green), respectively. *, reference strain. Distant genetic relationships are highlighted by red-colored connections and values.
(TIF)

S3 Fig The radii of satellitism. (A, B) *Av*. *paragallinarum* and bacteria of other species were cocultured on blood agar plates for 48 h, and the radii of satellitism generated by the *Av*. *paragallinarum* isolates 12-4N-1 (A) and X1-1S-1 (B) were measured. *P* values were determined by one-way ANOVA. The mean of radii is shown, and error bars represent the standard deviation (SD).
(TIF)

S4 Fig Antimicrobial phenotype and genotype of *Av*. *paragallinarum* and *S*. *chromogenes* isolates. The MICs of different antimicrobial agents to *Av*. *paragallinarum* (A) and *S*. *chromogenes* (B) were determined by the broth microdilution method. *Av*. *paragallinarum* isolates were serotyped through a mPCR assay to identify the serogroups. Antibiotic resistance genes in *Av*. *paragallinarum* and *S*. *chromogenes* isolates were identified using BLAST with a custom database. NA, nontypable. *, sequence identities of *tet(38)* in all isolates and *ant(6)-Ia* in the isolates *S*. *chromogenes* SC24, SC25, SC23, SC17, SC14 and SC22 were between 70% and 80%. Note: Multiple antibiotic-resistant phenotypes of most *Av*. *paragallinarum* were irrelevant to their genotypes. Similar to that of other Gram-negative bacteria, the outer membrane of *Av*. *paragallinarum* acts as a permeability barrier and prevents antibiotics from reaching their targets. In addition, resistance-mediating mutations are commonly seen in *Pasteurellaceae* of veterinary origin [67, 68]. Therefore, we hypothesized that *Av*. *paragallinarum* isolates may also evolve some gene mutations under the pressure of drugs and present different antimicrobial-resistant phenotypes.
(TIF)

S5 Fig Virulence genes carried by *S*. *chromogenes* and *Av*. *paragallinarum*. All *S*. *chromogenes* (n = 25) (A) and *Av*. *paragallinarum* isolates (n = 18) (B) were divided into two clusters with different colors (blue and green), respectively. Virulence genes were analyzed based on whole-genome sequencing and custom databases retrieved from VFDB (http://www.mgc.ac.cn/cgi-bin/VFs/compvfs.cgi?Genus=Haemophilus, http://www.mgc.ac.cn/cgi-bin/VFs/compvfs.cgi?Genus = Staphylococcus).
(TIF)

S6 Fig Growth curves of *Av*. *paragallinarum* and *S*. *chromogenes*. (A) Growth curves of *Av*. *paragallinarum* X1-1S-1 in the presence of 0.01% Triton, clindamycin (8 μg/mL), and vancomycin (32 μg/mL) for 24 h. The culture medium was TSB supplemented with 5% (v/v) serum and 0.0025% (w/v) NAD^+^. (B) Growth curves of *Av*. *paragallinarum* X1-1S-1 and *S*. *chromogenes* SC10 in the presence of 1 μM (E)-daporinad for 24 h. The culture medium for *Av*. *paragallinarum* was TSB supplemented with 5% (v/v) serum and 0.0025% (w/v) NAD^+^, and the culture medium for *S*. *chromogenes* was TSB supplemented with 5% (v/v) serum. The mean of six biological replicates is shown.
(TIF)

S7 Fig Different *Staphylococcus* species promote the survival of *Av*. *paragallinarum*. (A, B) Bacteria were cultured in TSB supplemented with 5% serum. Bacteria were cultured at 37°C (A) or 42°C (B). The mean of six biological replicates is shown. (C) High culture temperature promoted NAD^+^ production in *S*. *chromogenes*. *S*. *chromogenes* and *S*. *sciuri* were cultured in TSB supplemented with 5% serum for 4 h at 37°C or 42°C. *P* values were determined by one-way ANOVA. (D) *S*. *chromogenes* and *S*. *sciuri* showed no difference in promoting the survival of *Av*. *paragallinarum*. *P* values were determined by unpaired *t*-test. The mean of three biological replicates is shown, and error bars represent the standard deviation (SD) (n  =  3).
(TIF)

S8 Fig Water intake is greatly reduced when birds are coinfected with *S*. *chromogenes* and *Av*. *paragallinarum*. The water intake of each group was measured and recorded every morning before the renewal of drinking water.
(TIF)

S9 Fig Monoinfection of *S*. *chromogenes* hardly triggers clinical symptoms. The mean of six biological replicates is shown, and error bars represent the standard deviation (SD) (n  =  6).
(TIF)

S10 Fig *S.* *chromogenes* and *Av*. *paragallinarum* induce NAD^+^ production in host cells. (A) Replication rates of extracellular *Av*. *paragallinarum* compared to that of the initial inoculum. MH-S cells were infected with *Av*. *paragallinarum* X1-1S-1 (MOI = 1) alone or accompanied by *S*. *chromogenes* SC10 (MOI = 1) for 6 h. (B) *S*. *chromogenes* increased the number of extracellular *Av*. *paragallinarum*. MH-S or A549 cells were infected with *Av*. *paragallinarum* X1-1S-1 and/or *S*. *chromogenes* SC10 (MOI = 1) or incubated with 10 μg/mL vancomycin for 6 h. (C) *S*. *chromogenes* increased the abundance of extracellular NAD^+^. A549 cells were infected with *S*. *chromogenes* SC10 (MOI = 1) or incubated with 0.01% Triton or 1 μg/mL LPS for 6 h. (D) *S*. *chromogenes* impaired the integrity of A549 cells. A549 cells were infected with *S*. *chromogenes* SC10 (MOI = 1) or incubated with 0.01% Triton for 6 h. (E) LPS treatment produced no difference in the extracellular NAD^+^ content. MH-S cells were incubated with 1 μg/mL LPS for 6 h. (F) *S*. *chromogenes* and *Av*. *paragallinarum* increased the mRNA expression of *nampt* and *nmrk1*. A549 cells were infected with *S*. *chromogenes* SC10 and/or *Av*. *paragallinarum* X1-1S-1 (MOI = 1) or incubated with 10 μg/mL vancomycin for 6 h. *P* values were determined by unpaired *t*-test for (A, B, D and E) and one-way ANOVA for (C and F). The mean of three biological replicates is shown, and error bars represent the standard deviation (SD) (n  =  3).
(TIF)

S11 Fig (E)-Daporinad exhibits no cytotoxicity to respiratory cells. The cytotoxicity of (E)-daporinad to A549 and MH-S cells was determined through an LDH release assay. The mean of three biological replicates is shown, and error bars represent the standard deviation (SD) (n  =  3).
(TIF)

S12 Fig The extracellular growth of *Av*. *paragallinarum* is promoted by different *Staphylococcus* species. A549 cells were monoinfected with *Staphylococcus* spp. (MOI = 1) for 6 h to examine the mRNA expression of NAD^+^ synthetases, or A549 cells were coinfected with *Av*. *paragallinarum* X1-1S-1 and *Staphylococcus* spp. (MOI = 1) for 6 h to measure the number of extracellular *Av*. *paragallinarum*. *P* values were determined by one-way ANOVA. The mean of three biological replicates is shown, and error bars represent the standard deviation (SD) (n  =  3).
(TIF)

S13 Fig *Av*. *paragallinarum* enhances NAD^+^ biosynthesis in host cells. (A) *Av*. *paragallinarum* promoted the expression of NMRK1. A549 cells were infected with *S*. *chromogenes* SC10 and/or *Av*. *paragallinarum* X1-1S-1 (MOI = 1) for 6 h. *P* values were determined by one-way ANOVA. (B) *Av*. *paragallinarum* increased the intracellular NAD^+^ content. MH-S cells were infected with *Av*. *paragallinarum* X1-1S-1 (MOI = 1) for different times. *P* values were determined by unpaired *t*-test. The mean of three biological replicates is shown, and error bars represent the standard deviation (SD) (n  =  3).
(TIF)

S14 Fig *S*. *chromogenes* and *Av*. *paragallinarum* invade respiratory host cells. (A) Confocal images of internalized bacteria in epithelial cells. A549 cells were infected with pHrodoRed-labeled *Av*. *paragallinarum* X1-1S-1 or *S*. *chromogenes* SC10 (MOI = 20) for 1 h. F-actin was stained with ActinGreen 488 ReadyProbes (green), and the nuclei were counterstained with DAPI (blue). Scale bar = 10 μm. (B) The numbers of internalized *S*. *chromogenes* and *Av*. *paragallinarum* were measured by the gentamycin protection assay after the monoinfection or coinfection of these two bacteria (MOI = 1) for 6 h. (C) Merged exhibition of the extracellular and intracellular numbers of *Av*. *paragallinarum* from (B), Figs 6A and S10B. (D) Ratio of extracellular/intracellular *Av*. *paragallinarum* from (C). (E) Ratio of *Av*. *paragallinarum* in experimental groups of coinfection/monoinfection from (C). *P* values were determined by unpaired *t*-test. The mean of three biological replicates is shown, and error bars represent the standard deviation (SD) (n  =  3).
(TIF)
