# Supplementary figures and images for: Resident bacteria contribute to opportunistic infections of the respiratory tract
Source: PLoS Pathog. 2021 Mar 19;17(3):e1009436. doi: 10.1371/journal.ppat.1009436 (PMC8011790; doi:10.1371/journal.ppat.1009436)

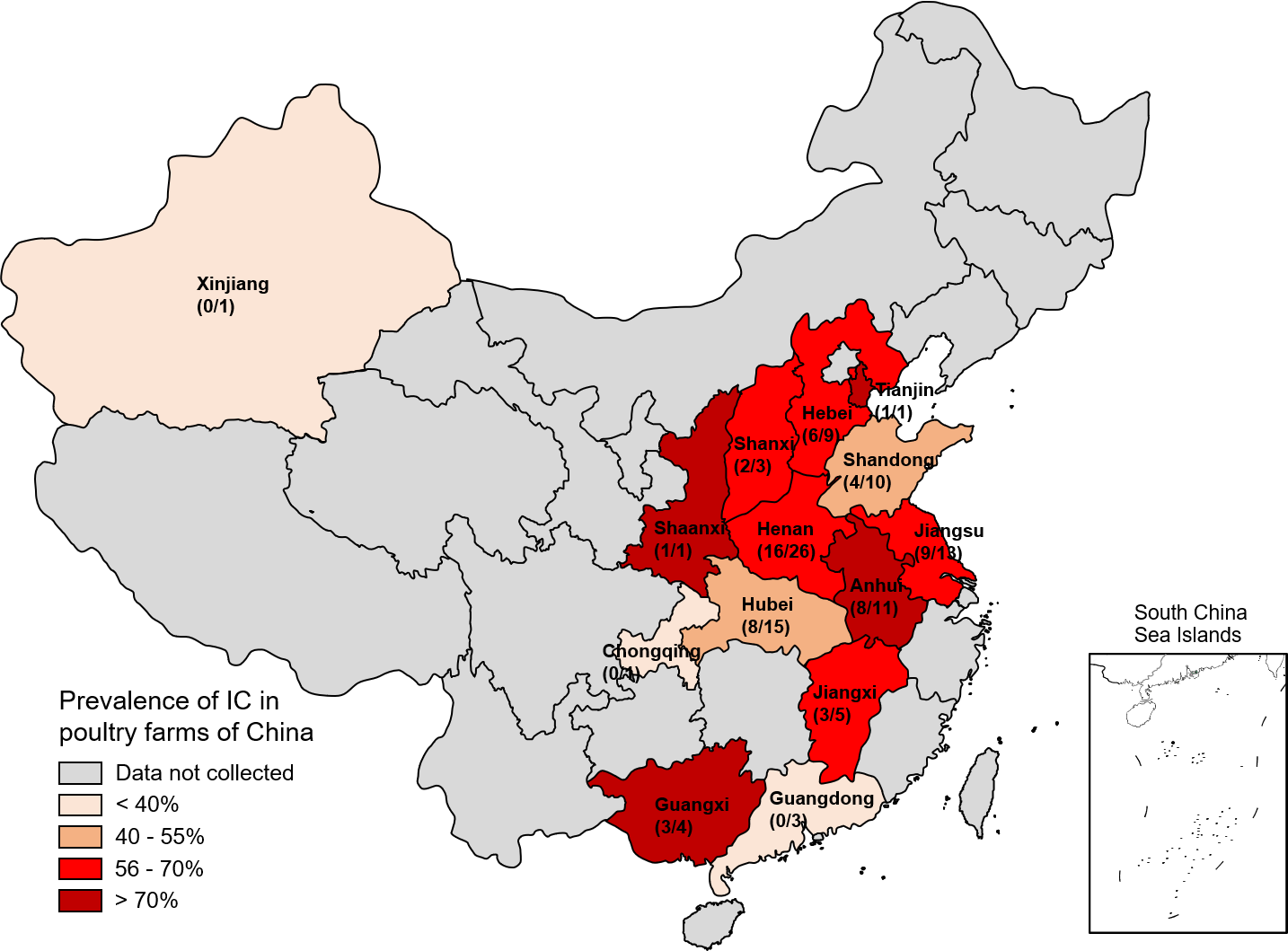

Supplement: S1 Fig — Prevalence of IC in China: data from 103 poultry farms were collected by questionnaires shared on the internet. Map layers were obtained from Wikimedia Commons (https://commons.wikimedia.org/wiki/File:China-outline.svg). (TIF) [file ppat.1009436.s009.tif]

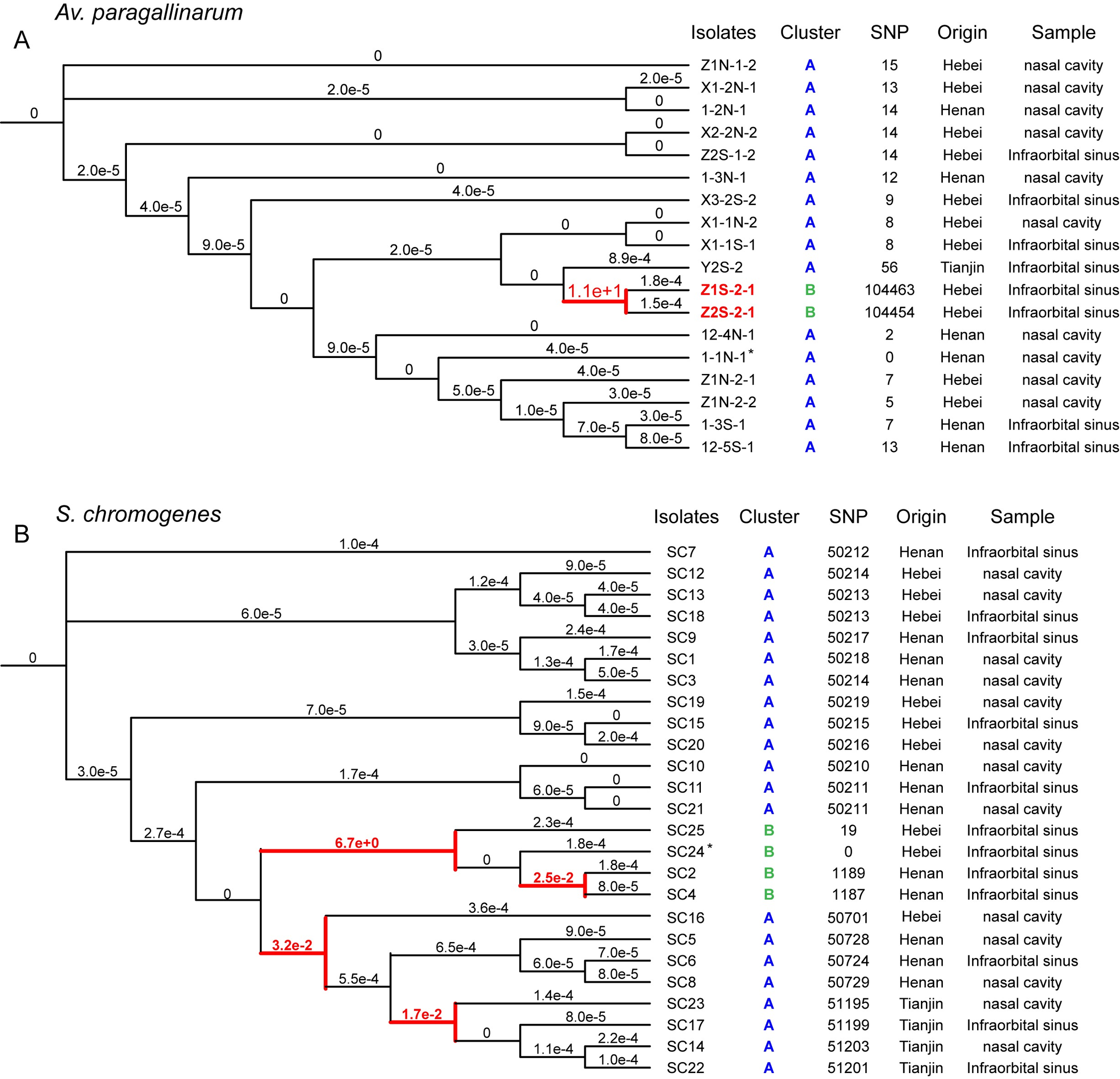

Supplement: S2 Fig — Core-genome SNP-based phylogenetic trees of Av. paragallinarum (A) and S. chromogenes (B). Bacteria of the two genera were divided into two genotypes (labeled with blue and green), respectively. *, reference strain. Distant genetic relationships are highlighted by red-colored connections and values. (TIF) [file ppat.1009436.s010.tif]

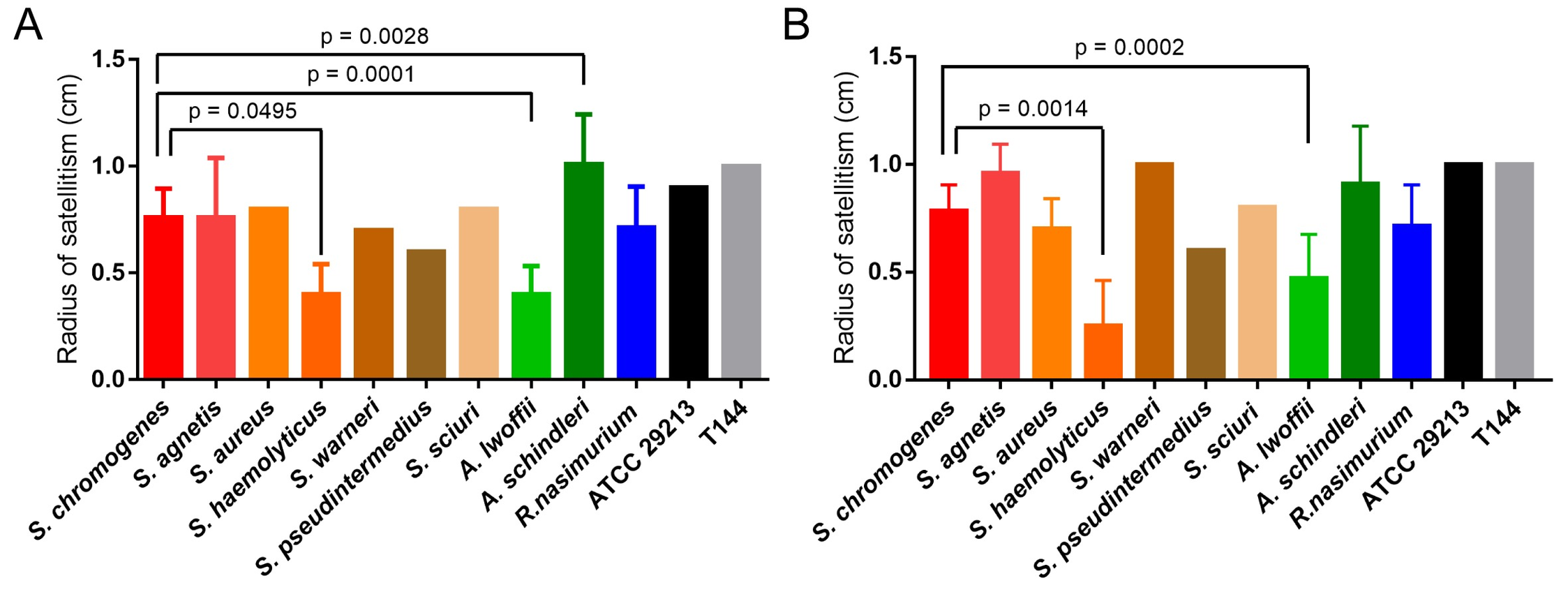

Supplement: S3 Fig — (A, B) Av. paragallinarum and bacteria of other species were cocultured on blood agar plates for 48 h, and the radii of satellitism generated by the Av. paragallinarum isolates 12-4N-1 (A) and X1-1S-1 (B) were measured. P values were determined by one-way ANOVA. The mean of radii is shown, and error bars represent the standard deviation (SD). (TIF) [file ppat.1009436.s011.tif]

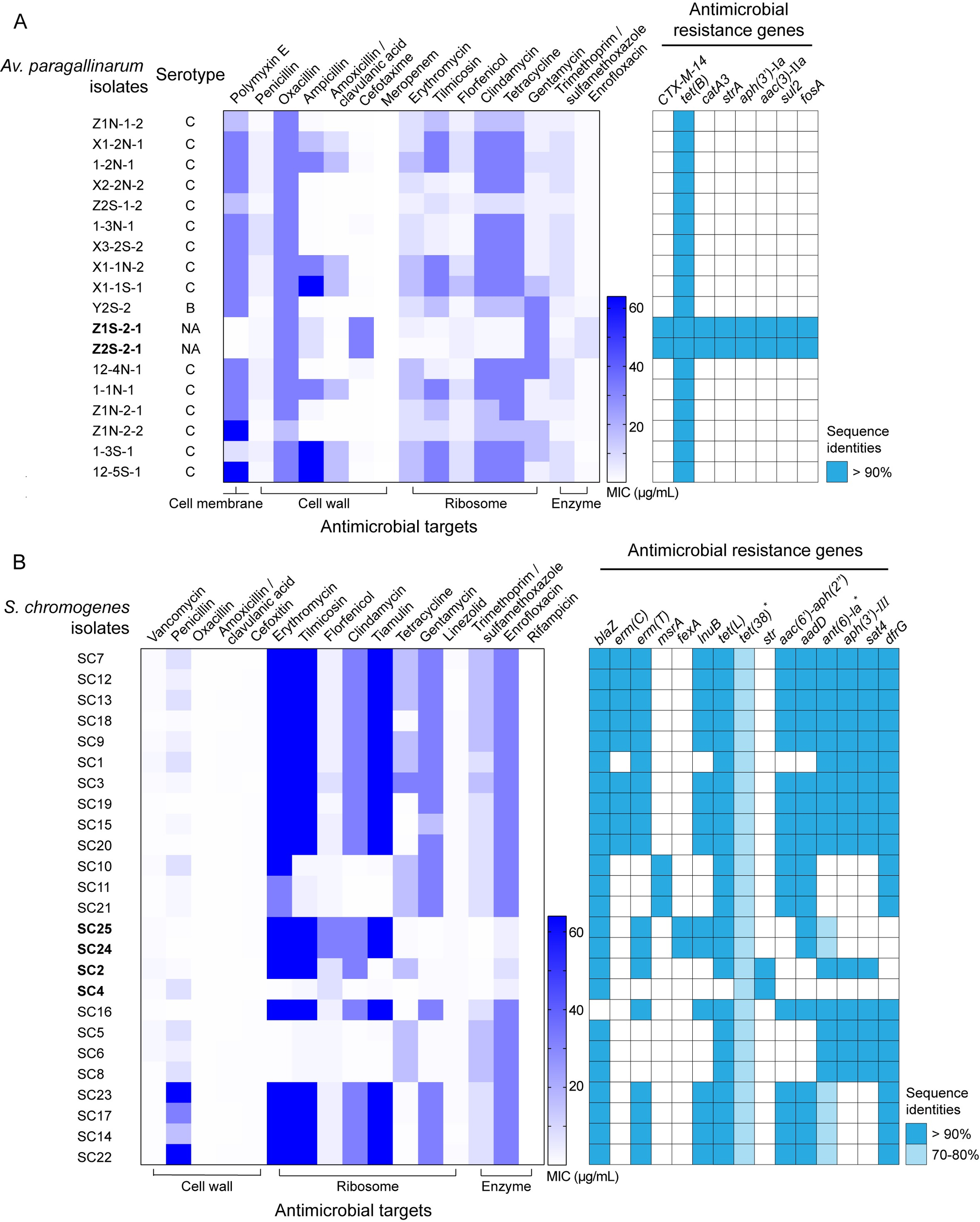

Supplement: S4 Fig — The MICs of different antimicrobial agents to Av. paragallinarum (A) and S. chromogenes (B) were determined by the broth microdilution method. Av. paragallinarum isolates were serotyped through a mPCR assay to identify the serogroups. Antibiotic resistance genes in Av. paragallinarum and S. chromogenes isolates were identified using BLAST with a custom database. NA, nontypable. *, sequence identities of tet(38) in all isolates and ant(6)-Ia in the isolates S. chromogenes SC24, SC25, SC23, SC17, SC14 and SC22 were between 70% and 80%. Note: Multiple antibiotic-resistant phenotypes of most Av. paragallinarum were irrelevant to their genotypes. Similar to that of other Gram-negative bacteria, the outer membrane of Av. paragallinarum acts as a permeability barrier and prevents antibiotics from reaching their targets. In addition, resistance-mediating mutations are commonly seen in Pasteurellaceae of veterinary origin [67, 68]. Therefore, we hypothesized that Av. paragallinarum isolates may also evolve some gene mutations under the pressure of drugs and present different antimicrobial-resistant phenotypes. (TIF) [file ppat.1009436.s012.tif]

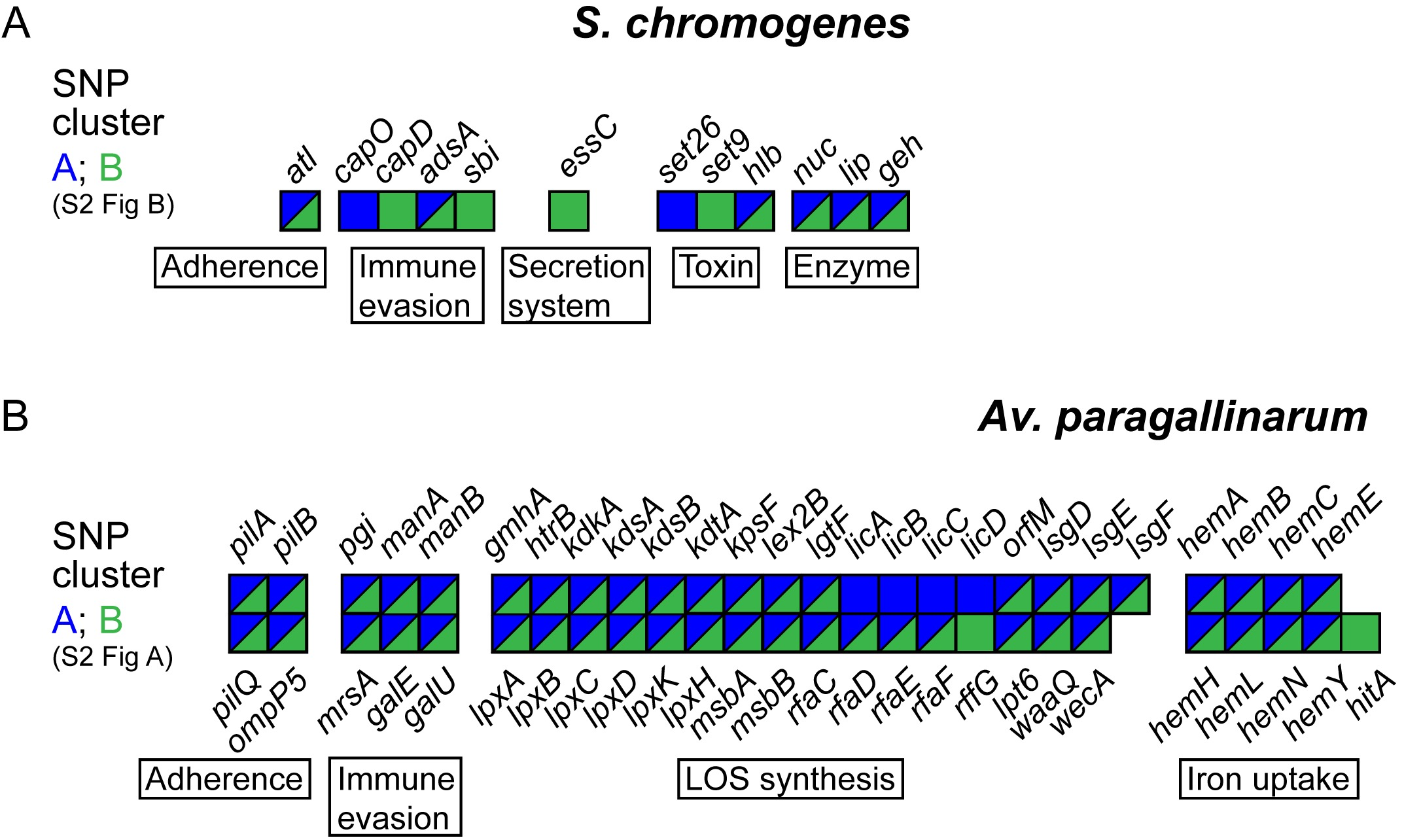

Supplement: S5 Fig — All S. chromogenes (n = 25) (A) and Av. paragallinarum isolates (n = 18) (B) were divided into two clusters with different colors (blue and green), respectively. Virulence genes were analyzed based on whole-genome sequencing and custom databases retrieved from VFDB (http://www.mgc.ac.cn/cgi-bin/VFs/compvfs.cgi?Genus=Haemophilus, http://www.mgc.ac.cn/cgi-bin/VFs/compvfs.cgi?Genus = Staphylococcus). (TIF) [file ppat.1009436.s013.tif]

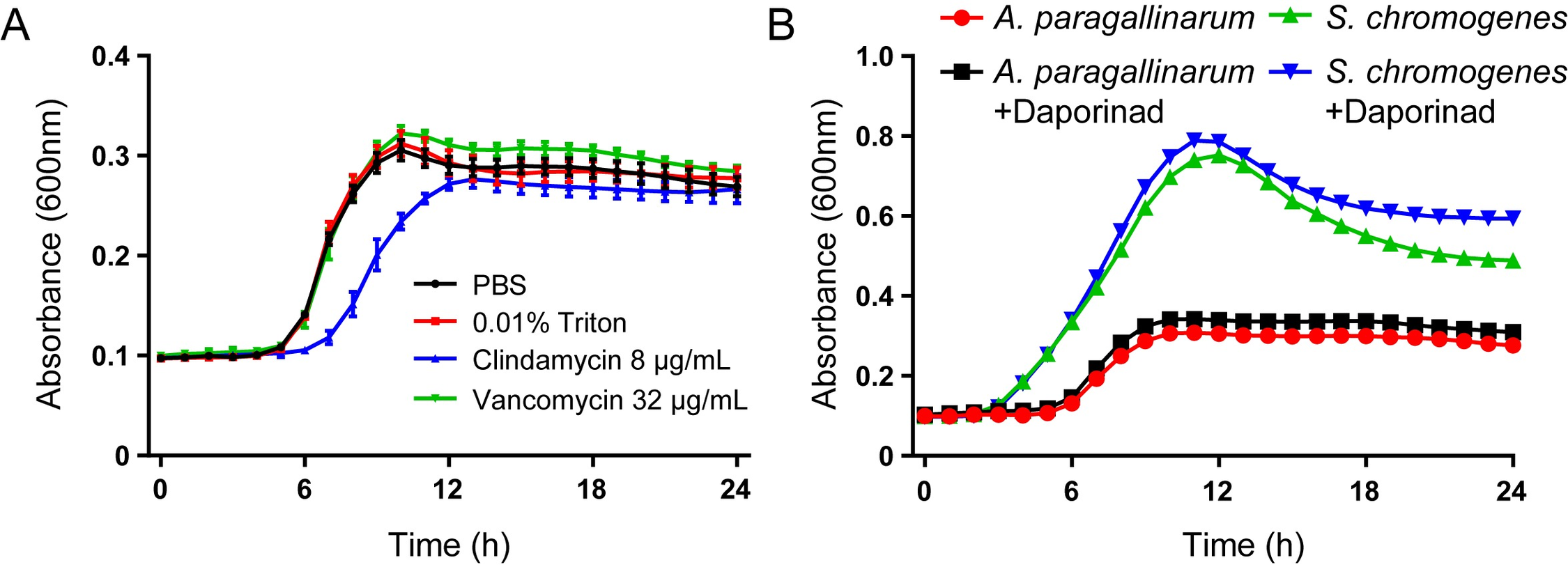

Supplement: S6 Fig — (A) Growth curves of Av. paragallinarum X1-1S-1 in the presence of 0.01% Triton, clindamycin (8 μg/mL), and vancomycin (32 μg/mL) for 24 h. The culture medium was TSB supplemented with 5% (v/v) serum and 0.0025% (w/v) NAD+. (B) Growth curves of Av. paragallinarum X1-1S-1 and S. chromogenes SC10 in the presence of 1 μM (E)-daporinad for 24 h. The culture medium for Av. paragallinarum was TSB supplemented with 5% (v/v) serum and 0.0025% (w/v) NAD+, and the culture medium for S. chromogenes was TSB supplemented with 5% (v/v) serum. The mean of six biological replicates is shown. (TIF) [file ppat.1009436.s014.tif]

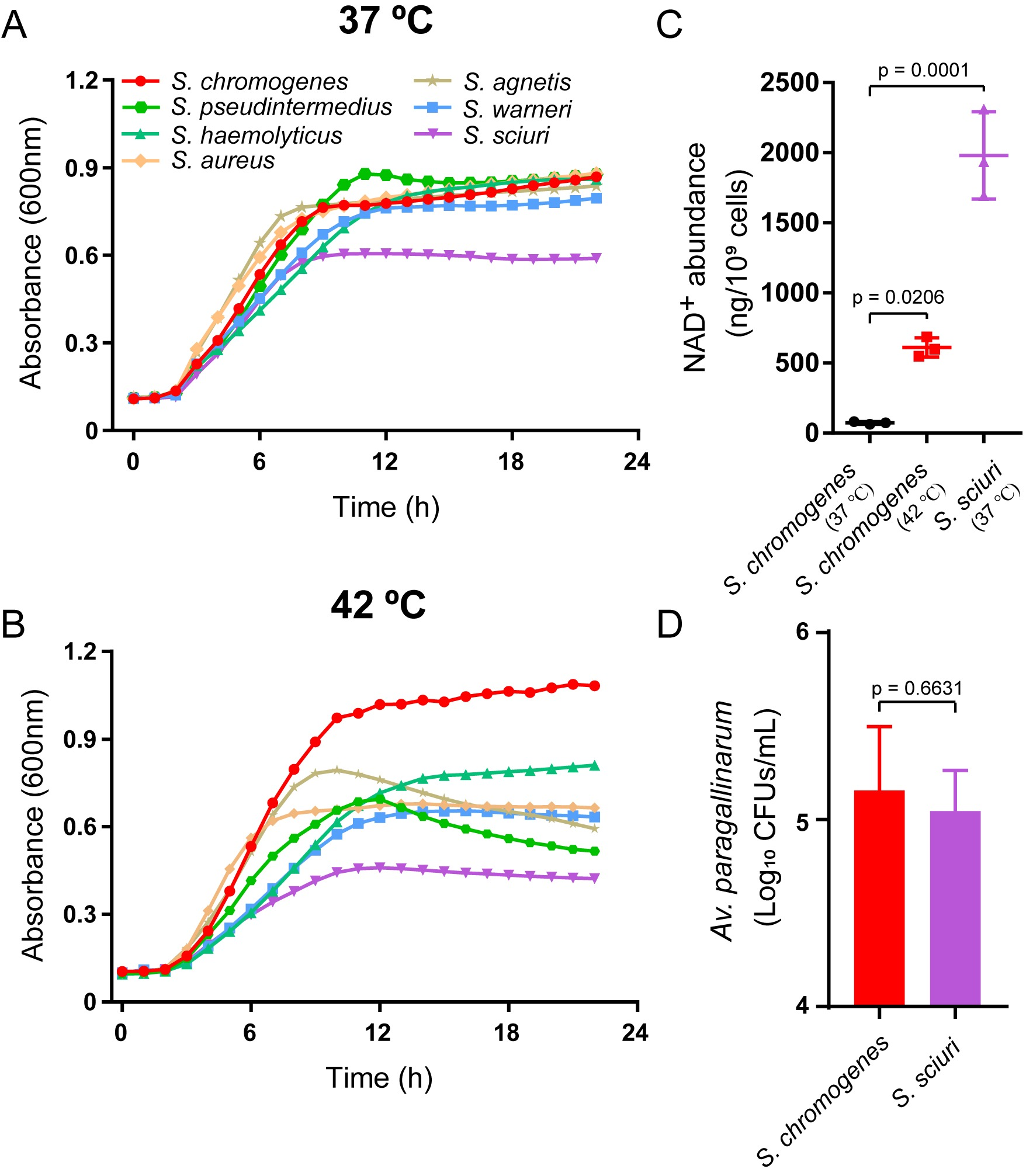

Supplement: S7 Fig — (A, B) Bacteria were cultured in TSB supplemented with 5% serum. Bacteria were cultured at 37°C (A) or 42°C (B). The mean of six biological replicates is shown. (C) High culture temperature promoted NAD+ production in S. chromogenes. S. chromogenes and S. sciuri were cultured in TSB supplemented with 5% serum for 4 h at 37°C or 42°C. P values were determined by one-way ANOVA. (D) S. chromogenes and S. sciuri showed no difference in promoting the survival of Av. paragallinarum. P values were determined by unpaired t-test. The mean of three biological replicates is shown, and error bars represent the standard deviation (SD) (n = 3). (TIF) [file ppat.1009436.s015.tif]

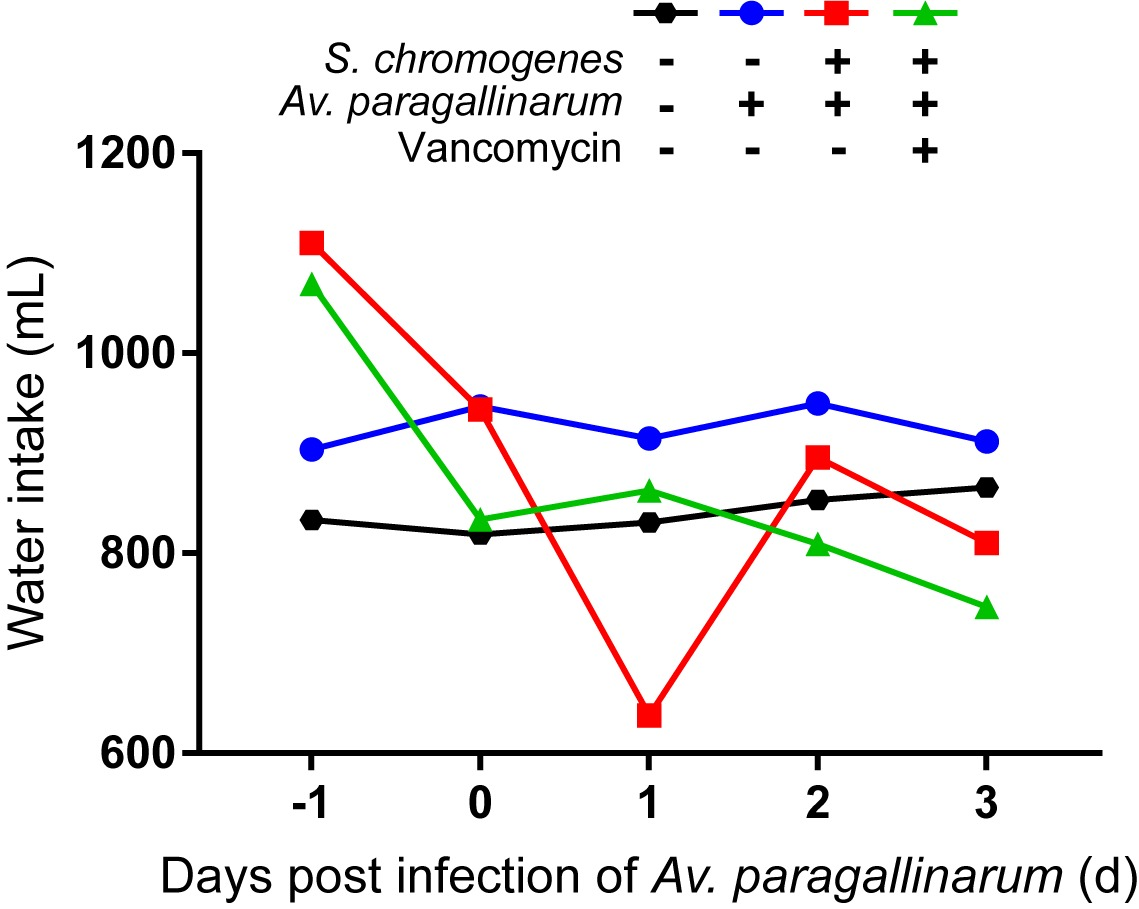

Supplement: S8 Fig — The water intake of each group was measured and recorded every morning before the renewal of drinking water. (TIF) [file ppat.1009436.s016.tif]

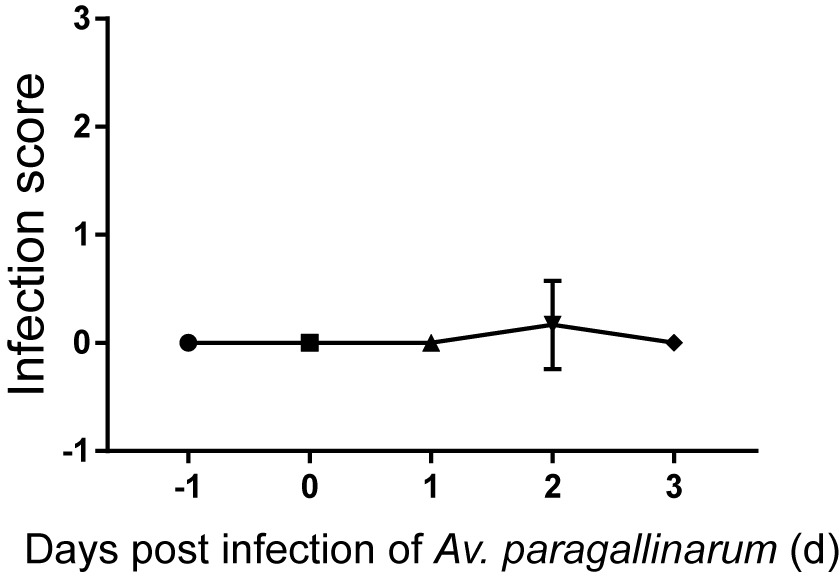

Supplement: S9 Fig — The mean of six biological replicates is shown, and error bars represent the standard deviation (SD) (n = 6). (TIF) [file ppat.1009436.s017.tif]

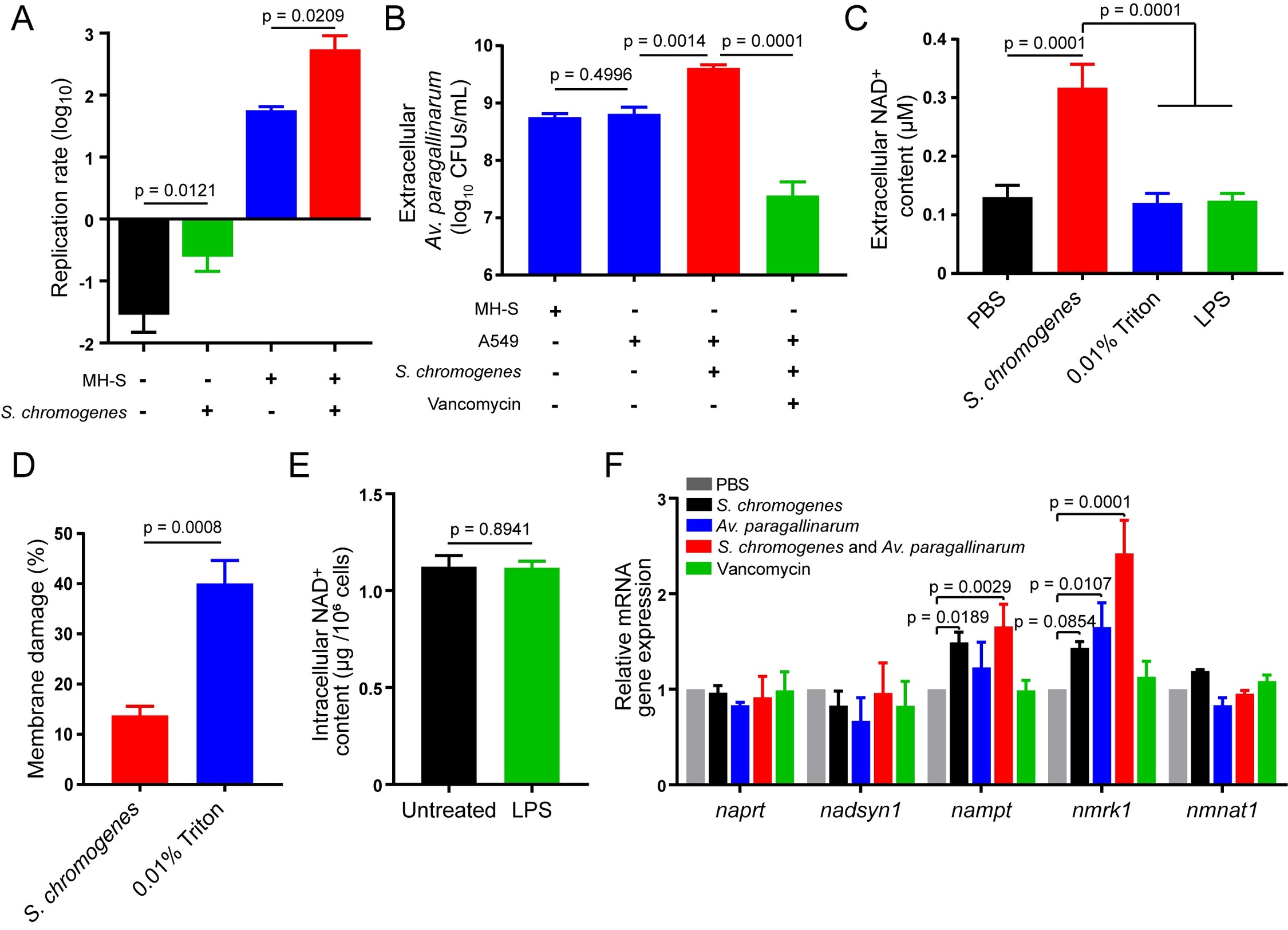

Supplement: S10 Fig — (A) Replication rates of extracellular Av. paragallinarum compared to that of the initial inoculum. MH-S cells were infected with Av. paragallinarum X1-1S-1 (MOI = 1) alone or accompanied by S. chromogenes SC10 (MOI = 1) for 6 h. (B) S. chromogenes increased the number of extracellular Av. paragallinarum. MH-S or A549 cells were infected with Av. paragallinarum X1-1S-1 and/or S. chromogenes SC10 (MOI = 1) or incubated with 10 μg/mL vancomycin for 6 h. (C) S. chromogenes increased the abundance of extracellular NAD+. A549 cells were infected with S. chromogenes SC10 (MOI = 1) or incubated with 0.01% Triton or 1 μg/mL LPS for 6 h. (D) S. chromogenes impaired the integrity of A549 cells. A549 cells were infected with S. chromogenes SC10 (MOI = 1) or incubated with 0.01% Triton for 6 h. (E) LPS treatment produced no difference in the extracellular NAD+ content. MH-S cells were incubated with 1 μg/mL LPS for 6 h. (F) S. chromogenes and Av. paragallinarum increased the mRNA expression of nampt and nmrk1. A549 cells were infected with S. chromogenes SC10 and/or Av. paragallinarum X1-1S-1 (MOI = 1) or incubated with 10 μg/mL vancomycin for 6 h. P values were determined by unpaired t-test for (A, B, D and E) and one-way ANOVA for (C and F). The mean of three biological replicates is shown, and error bars represent the standard deviation (SD) (n = 3). (TIF) [file ppat.1009436.s018.tif]

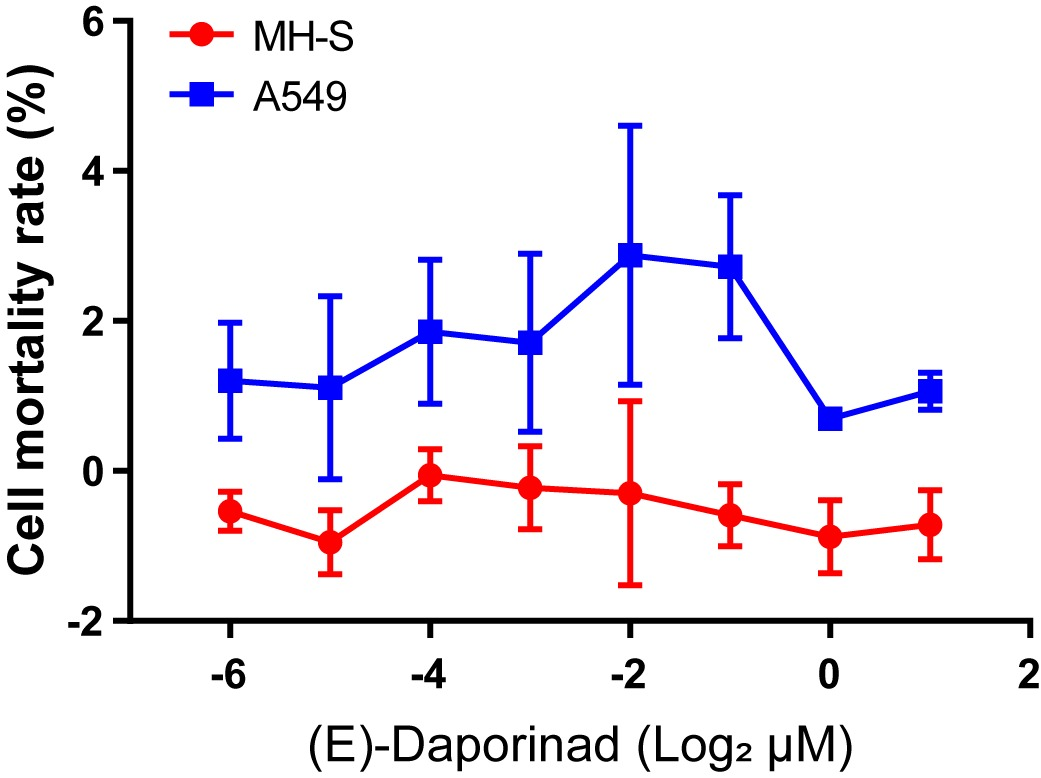

Supplement: S11 Fig — The cytotoxicity of (E)-daporinad to A549 and MH-S cells was determined through an LDH release assay. The mean of three biological replicates is shown, and error bars represent the standard deviation (SD) (n = 3). (TIF) [file ppat.1009436.s019.tif]

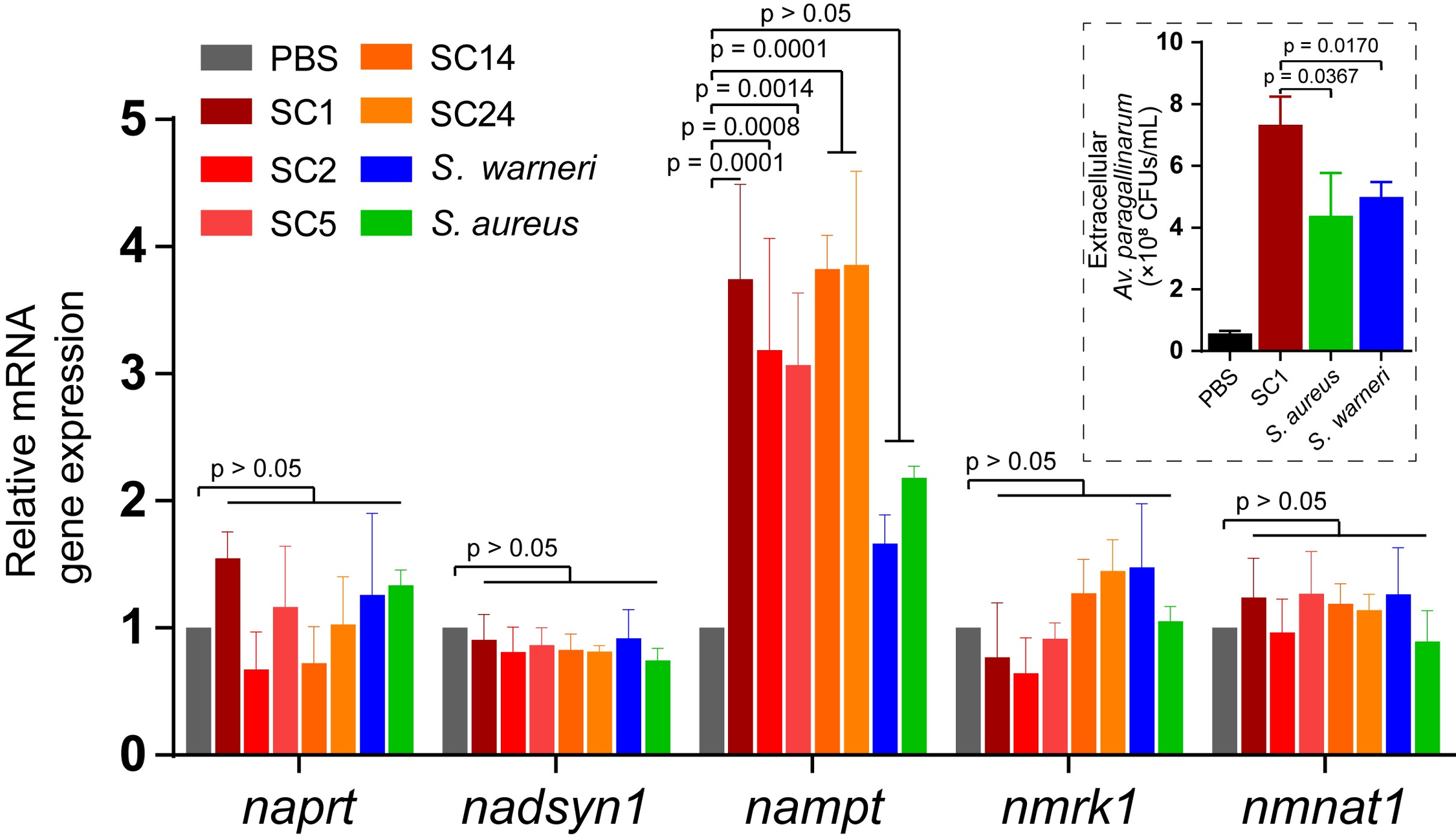

Supplement: S12 Fig — A549 cells were monoinfected with Staphylococcus spp. (MOI = 1) for 6 h to examine the mRNA expression of NAD+ synthetases, or A549 cells were coinfected with Av. paragallinarum X1-1S-1 and Staphylococcus spp. (MOI = 1) for 6 h to measure the number of extracellular Av. paragallinarum. P values were determined by one-way ANOVA. The mean of three biological replicates is shown, and error bars represent the standard deviation (SD) (n = 3). (TIF) [file ppat.1009436.s020.tif]

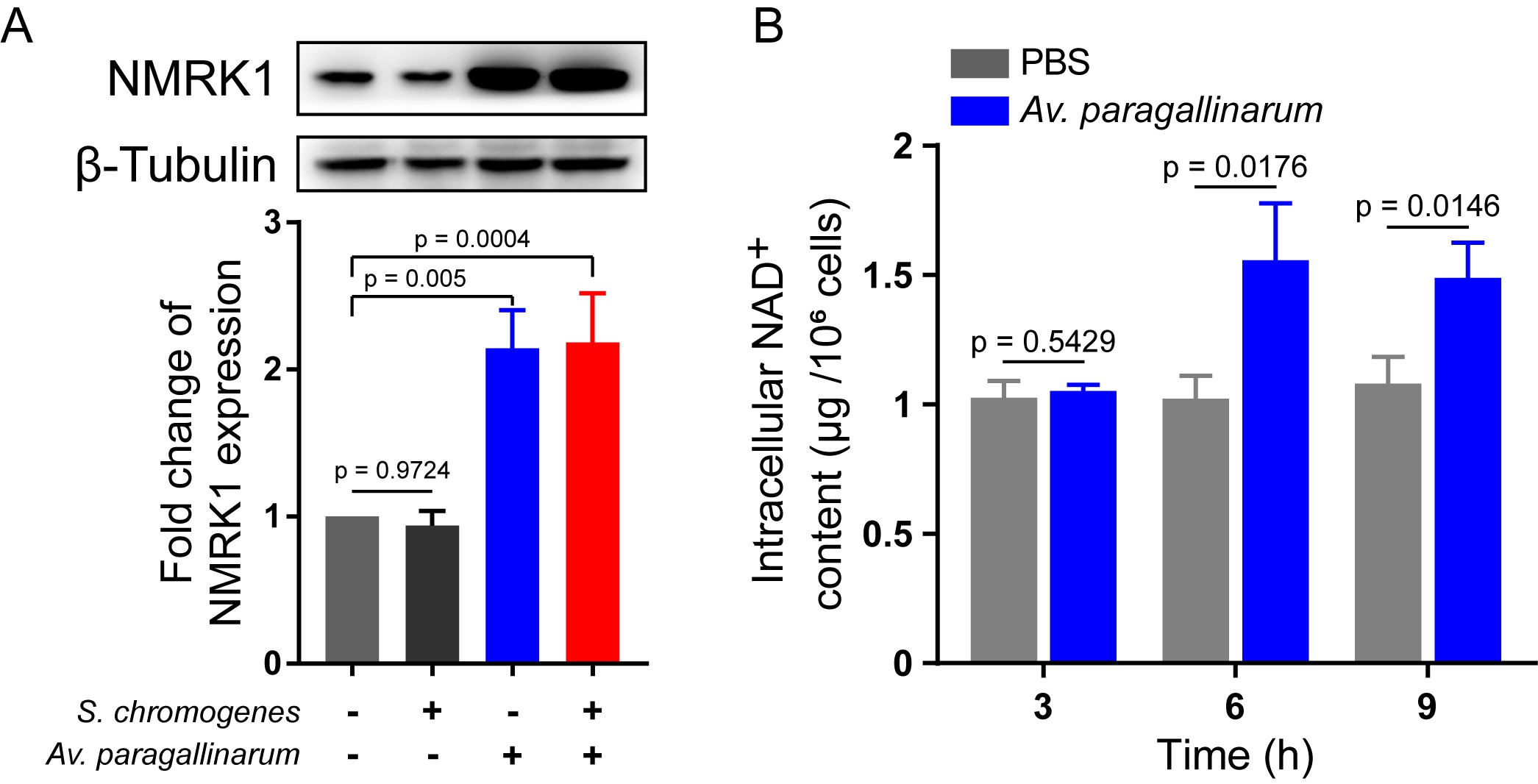

Supplement: S13 Fig — (A) Av. paragallinarum promoted the expression of NMRK1. A549 cells were infected with S. chromogenes SC10 and/or Av. paragallinarum X1-1S-1 (MOI = 1) for 6 h. P values were determined by one-way ANOVA. (B) Av. paragallinarum increased the intracellular NAD+ content. MH-S cells were infected with Av. paragallinarum X1-1S-1 (MOI = 1) for different times. P values were determined by unpaired t-test. The mean of three biological replicates is shown, and error bars represent the standard deviation (SD) (n = 3). (TIF) [file ppat.1009436.s021.tif]

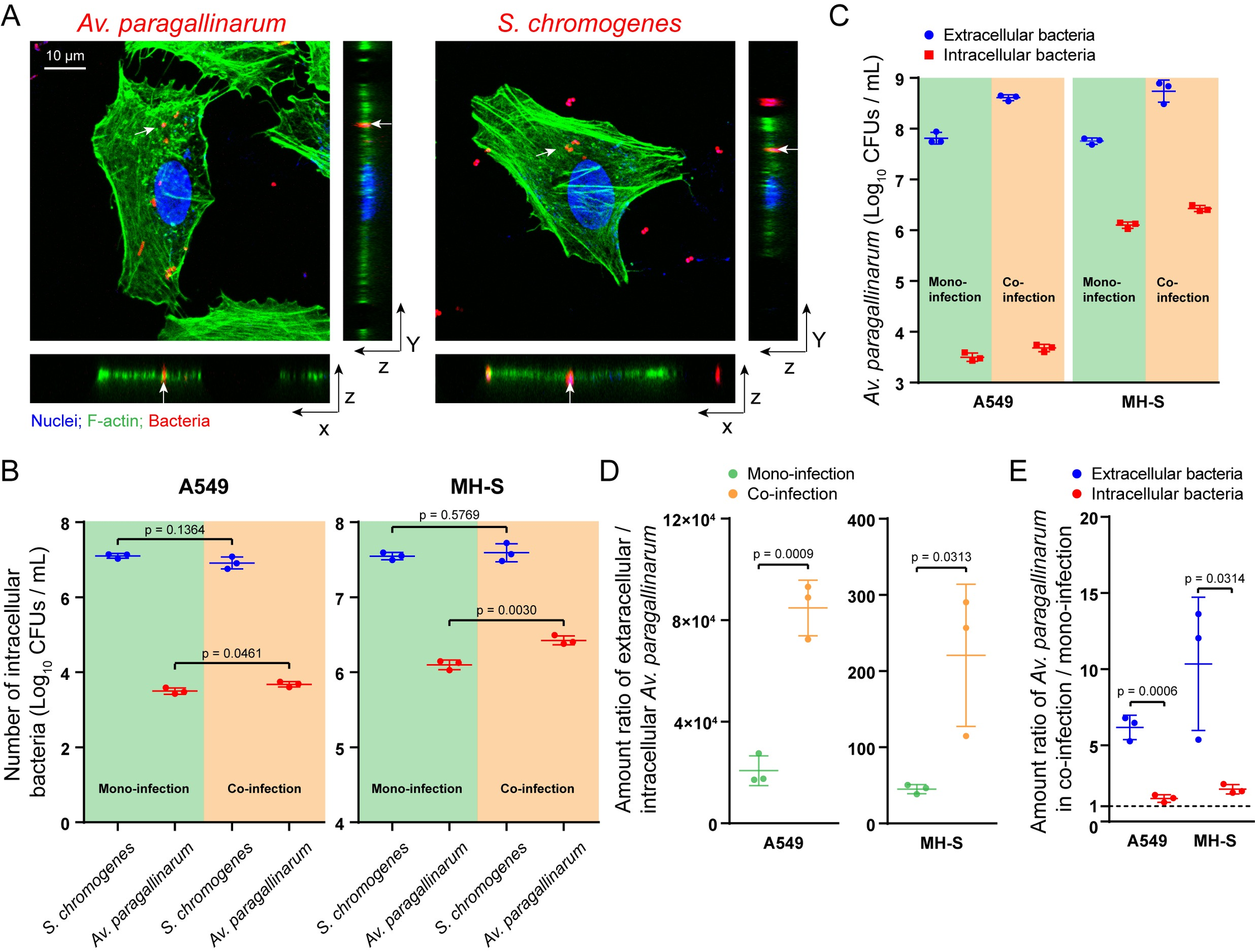

Supplement: S14 Fig — (A) Confocal images of internalized bacteria in epithelial cells. A549 cells were infected with pHrodoRed-labeled Av. paragallinarum X1-1S-1 or S. chromogenes SC10 (MOI = 20) for 1 h. F-actin was stained with ActinGreen 488 ReadyProbes (green), and the nuclei were counterstained with DAPI (blue). Scale bar = 10 μm. (B) The numbers of internalized S. chromogenes and Av. paragallinarum were measured by the gentamycin protection assay after the monoinfection or coinfection of these two bacteria (MOI = 1) for 6 h. (C) Merged exhibition of the extracellular and intracellular numbers of Av. paragallinarum from (B), Figs 6A and S10B. (D) Ratio of extracellular/intracellular Av. paragallinarum from (C). (E) Ratio of Av. paragallinarum in experimental groups of coinfection/monoinfection from (C). P values were determined by unpaired t-test. The mean of three biological replicates is shown, and error bars represent the standard deviation (SD) (n = 3). (TIF) [file ppat.1009436.s022.tif]
